# A new method for the comparison of ^1^H NMR predictors based on tree-similarity of spectra

**DOI:** 10.1186/1758-2946-6-9

**Published:** 2014-03-25

**Authors:** Andrés M Castillo, Andrés Bernal, Luc Patiny, Julien Wist

**Affiliations:** 1 Facultad de Ingeniería, Universidad Nacional de Colombia, Bogotá, DC, Colombia; 2 Grupo de Química Teórica, Universidad Nacional de Colombia, Bogotá, DC, Colombia; 3 Institute of Chemical Sciences and Engineering, Ecole Polytechnique Fédérale de Lausanne (EPFL), CH-1015 Lausanne, Switzerland; 4 Chemistry Department, Universidad del Valle, AA 25360 Cali, Valle, Colombia

**Keywords:** Nuclear magnetic resonance, Spectrum prediction, Chemical shift prediction, Spectral similarity, Trees

## Abstract

A methodology based on spectral similarity is presented that allows to compare NMR predictors without the recourse to assigned experimental spectra, thereby making the task of benchmarking NMR predictors less tedious, faster, and less prone to human error. This approach was used to compare four popular NMR predictors using a dataset of 1000 molecules and their corresponding experimental spectra. The results found were consistent with those obtained by directly comparing deviations between predicted and experimental shifts.

## Background

Cheminformatics plays an increasingly important role in structure validation by NMR spectroscopy, providing methods and algorithms for computer-assisted NMR spectra assignment and structure elucidation [1-8], as well as prediction and simulation [9-19] of spectra. Those methods heavily rely on the accuracy of predicted NMR parameters and thus along with the introduction of such novel methods comes the need to compare and evaluate available predictors. The established approach for this task consists in comparing the predicted NMR parameters, i.e., chemical shifts and coupling constants, with experimentally determined ones. Such approach comports the need to manually assign experimental data. As an alternative, benchmarking of NMR predictors could be performed using techniques of cheminformatics itself, avoiding errors due to manual assignment.

In a recent article the authors presented a tree-based method for measuring similarity between NMR spectra [20]. It was shown to produce results comparable to those of the binning method [21], with significant improvement in efficiency by focusing on the regions of the spectrum containing most of the information. Furthermore, this new approach directly operates on raw spectra, i.e., doesn’t relies on peak-picking. These features turn it into an attractive tool for the comparison and evaluation of NMR predictors, as it allows to measure the similarity between predictions and experiments without having recourse to assigned spectra. This article presents such a methodology and validates it against the established approach, for four common predictors.

The success of an NMR prediction algorithm is determined by its ability to reproduce the experimental chemical shifts. Determining the adequacy of a prediction thus implies having assigned the experimental spectra, and having their chemical shifts compared with predicted ones. Peak-picking and assignment are troublesome and time-demanding tasks, however. As an alternative, we propose to evaluate the success of a prediction algorithm by its ability to produce, by means of a proper simulation algorithm, a spectrum that is *sufficiently* similar to the one given by the experiment. The meaning of *sufficiently* similar will be discussed later in the text in the *Methods* section.

## Results and discussion

**Figure 1:**
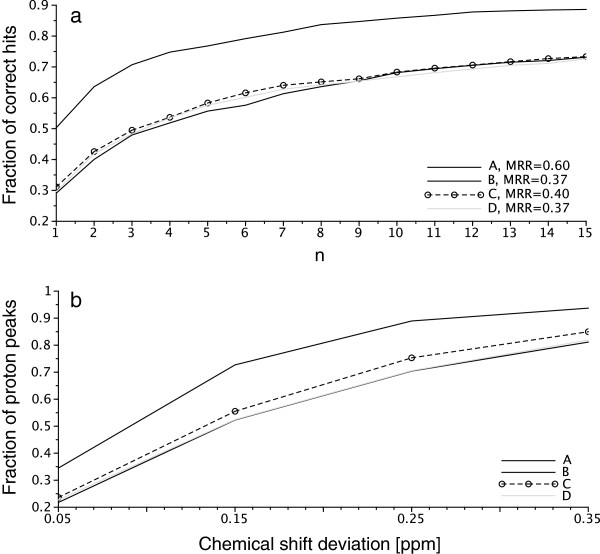
**Comparison of four commercially available predictors. a)** Results of the evaluation of 4 ^1^H NMR predictors using the new methodology. Each point in the plot corresponds to the fraction of correct matches within the n highest-ranking hits of a query of 1000 simulated ^1^H spectra to the database of the corresponding experimental spectra. For example, using predictor A, around 75% of the correct matches are found within the 4 highest ranked hits. Higher curves then represent better performance. Overall MRRs obtained for each predictor are specified in the legend. **b)** Results of the evaluation using direct comparison of predicted and observed chemical shifts. Each point in the plot corresponds to the fraction of predicted chemical shifts that fall within the specified deviation from the observed shift. For example, using predictor A, around 75% of the predicted peaks fall within 0.15 ppm of the observed peaks. Higher curves then represent better performance.

Figure 1a shows the distributions of correct matches within the *n* highest-ranked hits for each prediction algorithm. It can be observed that predictor A performed significantly better than all other algorithms. This result is confirmed by the Mean Reciprocal Rank (MRR) values. To validate our approach, we repeated the ranking of the four prediction tools but using a *traditional* approach: experimental signals were assigned to their corresponding nuclei and the differences between experimental and predicted shifts were computed. These chemical shift errors were partitioned on 0.1 ppm intervals up to 0.35 ppm, a value that already comprises over 90% of the predictions for the best performing method and over 80% for all predictors evaluated.

The resulting histograms are also shown in Figure 1b. Again, the performances of predictor A were found superior, producing around 10% more predictions on the two lower error intervals, while the other systems performed similarly, in agreement with the results obtained using our method.

**Figure 2:**
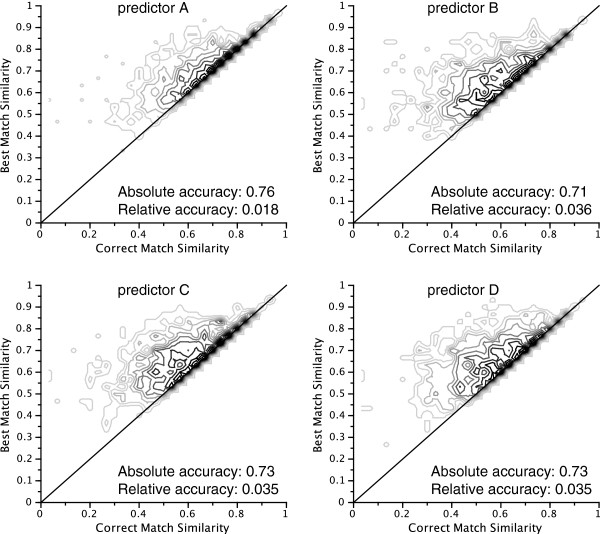
**Contour plots on the best match vs. correct match similarity plane of the query distributions.** An ideal prediction tool would have all the density packed along the diagonal.

Figure 2 displays the queries associated with each of the predictors on the correct match similarity vs. best match similarity plane. Clearly, queries that used predictor A are more closely packed along the identity line, which is associated with better relative accuracy as discussed in the section *Methods*. This is confirmed by computation of the mean relative prediction accuracy (see Figure 2). The remaining three predictors were found to perform similarly, thus reproducing the ranking given by the MRRs and corroborating that these results are not biased by the similarity measure.

## Experimental

A set of 1000 molecules of up to 33 heavy atoms was randomly selected from the Maybridge catalogue [22] (see Additional file 1) and the corresponding ^1^H NMR spectra kindly provided by Maybridge (see Additional file 2). The spectra were acquired with a 250 MHz Bruker spectrometer using a standard bruker pulse sequence (zg30), a relaxation delay of 1 s, a 30° excitation pulse at 27 kHz and an observation window of 20.693 ppm centered at 6.175 ppm. Each spectrum was binned and stored as a 1024 real points vector. For each molecule, the proton chemical shifts were predicted using four different prediction tools, referred to as A, B, C and D. The original spectra (1024 point, jcamp format), the raw predictions and a matrix of simulated spectra of 1024 points are provided in Additional files 3 and 4. We decided to keep predictors anonymous to maintain the focus of this work on the method to rank predictors, rather than the ranking itself. Each prediction was used to simulate a 1024 point spectrum at a frequency of 250 MHz with an algorithm that we described elsewhere [19]. Similarity matrices between simulated and experimental spectra, MRR, and average absolute and relative prediction accuracy were computed for each data set using the methodology described in the previous Section. A subset of 298 randomly chosen molecules were manually assigned in order to perform the evaluation by direct comparison of predicted and observed chemical shifts and compare the results obtained with those produced by our method. A subset was used for this part due to time constraints.

## Conclusions

The direct comparison of simulated and experimental spectra using an adequate similarity measure allows for an efficient and fully automatic methodology to evaluate NMR prediction algorithms. Results obtained using this new method are consistent with those obtained by the traditional chemical shift comparison method, but without the need for peak-picking and assignment. We therefore provide a method that can help improving NMR predictors in the future by allowing the comparison of predictors using datasets that are too large to be assigned manually.

## Methods

**Figure 3:**
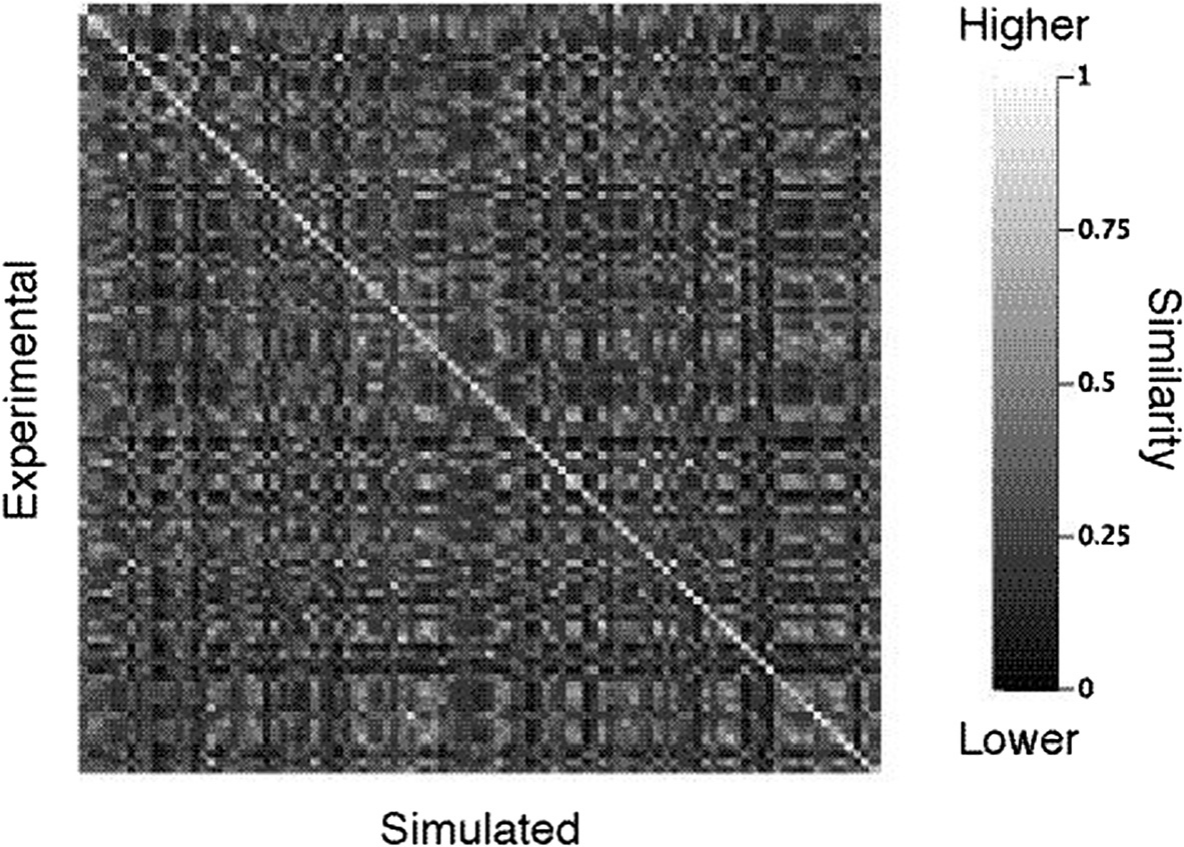
**Example of a spectra similarity matrix.** Rows correspond to experimental spectra and columns to simulated spectra of a 100 molecules data set, matrix elements give the similarity between the corresponding experimental and simulated spectrum. The thin light gray line on the diagonal shows a trend towards higher similarity between the matching spectra, as expected for an accurate NMR predictor.

To illustrate what is understood by *sufficiently* similar, we consider the experimental and simulated spectra for each element of a collection of molecules and build the matrix of similarities between each experimental and each simulated spectrum (see Figure 3). An accurate prediction algorithm would ensure that the highest similarity values lay on the diagonal of such matrix, i.e. the experimental spectrum of any given molecule would be more similar to its simulation than to simulated spectra of other molecules.

Now, consider a query of the experimental spectrum of some given molecule to a database of simulated spectrum. The result of the query is a list of database entries sorted in decreasing order of similarity to the experimental spectrum. For each query, the rank of a match is defined as the position of the matched simulated spectrum in this list. The more accurate the prediction, the better the rank of the simulated spectrum corresponding to the target molecule. The average performance of a predictor over a large set of queries can thus be measured by its Mean Reciprocal Rank (MRR),

$$\text{MRR}=\frac{1}{n}\sum_{i=1}^{n}\frac{1}{{\text{rank}}_{i}}$$

where *n* is the number of queries and *rank*_*i*_ is the rank of the correct match in the i-th query.

**Figure 4:**
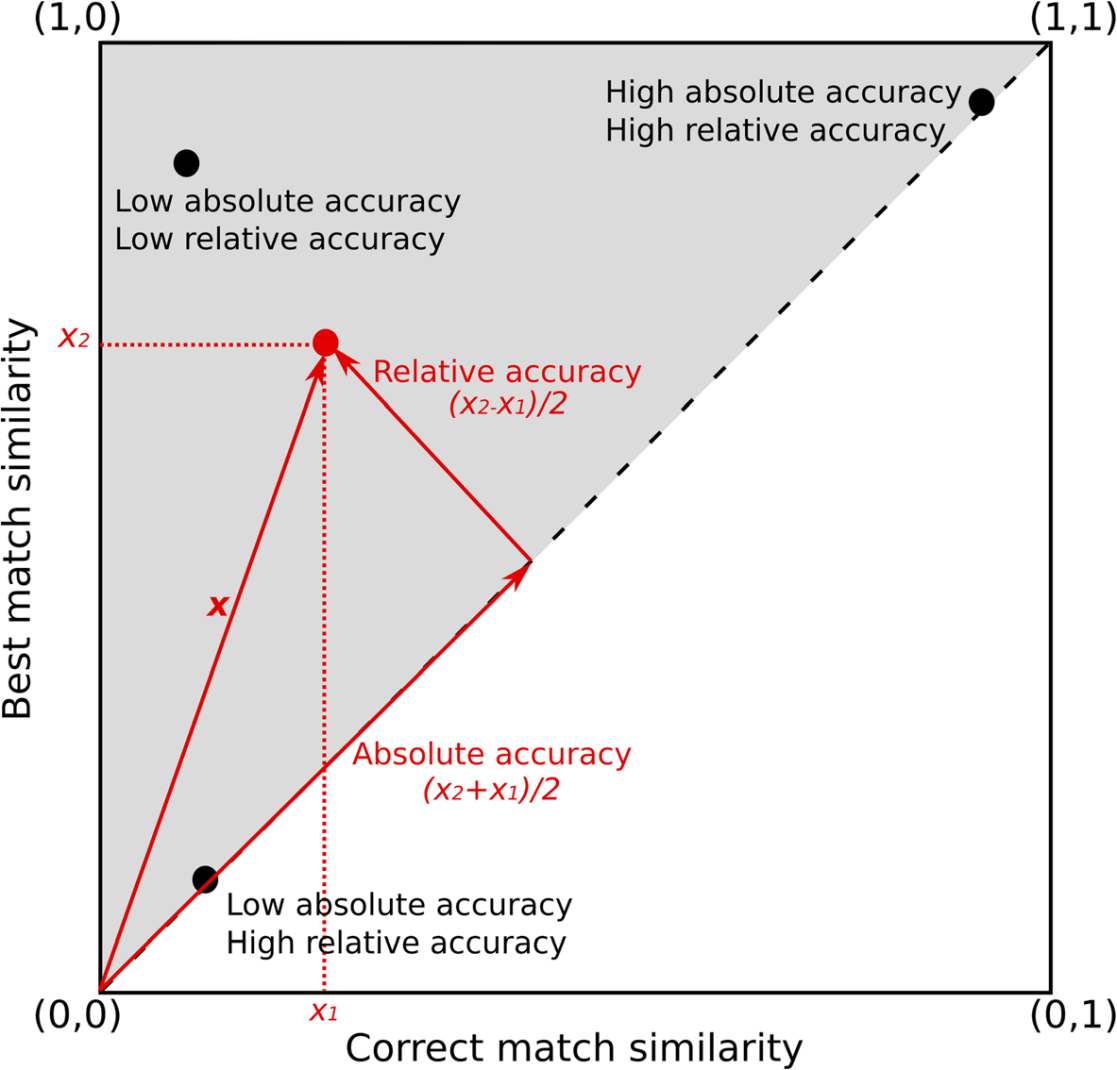
**Correct-match-similarity vs. best-match-similarity plane.** Black dots represent queries of simulated spectra to a database of experimental ones. The position of each query can be described in terms of two orthogonal vectors; one related to the absolute accuracy of the prediction, other to the inaccuracy relative to the dataset.

Note that the MRR ignores the actual similarity values computed. This is intentional, as we are not interested in how exact are similarities between correct matches, but on whether the prediction algorithm is able to generate a spectrum that can be unequivocally distinguished as that of the input molecule. However, a low-ranking correct match may be due not to poor prediction but to poor resolution of the similarity measure, which would lead to large sets of alternatives equally similar to the query spectrum. In such case, it would be the similarity measure, rather than the prediction, that fails at discriminating the correct match. To ensure that results are not biased by issues of the similarity measure, we propose a complementary approach that associates each query with a point in the correct-match-similarity vs. best-match-similarity plane (see Figure 4). In this plane:

• All queries are located on the upper triangle (gray area), as the similarity measure ranges from 0 to 1.

• Points located on the diagonal (dotted line) correspond to those cases where the best match is the correct match.

• The accuracy of the prediction in absolute terms (i.e. in terms of the similarity between the correct match and the experimental spectrum) increases as we move up to the extreme at (1,1), where the correct match and the experimental spectrum are identical. We then refer to the magnitude of the component of the query on this direction as the *absolute prediction accuracy* (see Figure 4).

• The accuracy of the prediction relative to the data set (i.e. the ratio between the experimental spectrum’s similarity to the correct match and its similarity to the best match in the data set) decreases as we move away from the identity line. We then refer to the magnitude of the component of the query vector orthogonal to the absolute prediction accuracy as the *relative prediction accuracy* (see Figure 4).

Low relative prediction accuracy means that the correct match is as similar or almost as similar to the queried spectrum as the best match in the whole database. Good predictions can then be associated with low values of relative accuracy. Note that this approach looks into the actual similarity values between the experimental and simulated spectra regardless of the rank of the correct match, which is exactly the opposite of what we achieved with the MRR. Combining the two approaches we can distinguish between low-ranking queries due to poor prediction and low-ranking queries due to an inadequate similarity measure: as long as the same trends result from evaluating performance in terms of the relative accuracy index or in terms of the MRR, we can be certain that the evaluation is not biased by a poorly discriminating similarity measure.

It follows from the previous discussion that the choice of an appropriate similarity measure is key to the success of the methodology proposed. Here we used the tree-based methodology that has been described in detail elsewhere [20]. In brief, it consists in building a tree representation of each spectrum that summarizes key information on its signal-rich regions, followed by the computation of a similarity measure between these trees. This similarity measure is defined recursively, so that the similarity between two trees at depth *k* depends on the similarity between nodes located on that level, and on the similarity between the trees at depth *k + 1*. This technique is similar to the traditional binning technique [21], but presents the advantage of focusing on regions with high signal intensity, using fewer data points by avoiding large blank or merely noisy zones.

## Competing interests

The authors declare that they have no competing interests.

## Authors’ contributions

AMC conceived this study, while LP and JW supervised its design and execution. AMC developed the code with the help of LP and AB drafted the manuscript with the help of JW. JW and AB achieved all illustrations jointly. All authors participated in the redaction of this manuscript and approved it.

## Supplementary Material

Additional file 1 **File containing the molecules in .mol format.** Description of data: A set of 1000 molecules that can be used to benchmark nmr predictors with molecular weight up to 33 heavy atoms. These molecules were picked randomly from the maybridge catalogue (http://www.maybridge.com/). Click here for file

Additional file 2 **Experimental spectra corresponding to the molecules of the molfile.sdf.zip.** This file contains the set of standard proton spectra acquired at 250 MHz and kindly provided by Maybridge (http://maybridge.com). The original spectra were binned and stored in a matrix as Y vectors of 1024 points ordered according to the molfile.sdf.zip file. Click here for file

Additional file 3 **Directory containing the source code of the algorithm described above and that allows to compute a similarity matrix such as depicted in Figure** 3**.** A directory that contains the source code used in this work, compiled classes, a compiled version of the code (jar file) and all the necessary input data in order to replicate the results of Figure 1A, including the original predictions obtained with the four predictors. A Readme.txt file explains the content of this directory in more details. Click here for file

Additional file 4 **Directory containing a graphical tool to benchmark new predictions (submitted as input file in the correct format) with the predictions shown in this publication.** The graphical tool provided in this compressed archive allows to compute and visualize the results for a new input file containing a new set of predictions. It consist in a web page (index.html) that can be accessed locally or remotely if the directory is placed on a server. The input file is simply *drag and dropped* on the webpage in order to start the computation of the complete similarity matrix and on the different statistical indicator including the curve of Figure 1. Click here for file
