# Supplementary material for: A new method for the comparison of 1H NMR predictors based on tree-similarity of spectra
Source: J Cheminform. 2014 Mar 25;6:9. doi: 10.1186/1758-2946-6-9 (PMC3987679; doi:10.1186/1758-2946-6-9)
Supplement: Additional file 4 — Directory containing a graphical tool to benchmark new predictions (submitted as input file in the correct format) with the predictions shown in this publication. The graphical tool provided in this compressed archive allows to compute and visualize the results for a new input file containing a new set of predictions. It consist in a web page (index.html) that can be accessed locally or remotely if the directory is placed on a server. The input file is simply drag and dropped on the webpage in order to start the computation of the complete similarity matrix and on the different statistical indicator including the curve of Figure 1. [file 1758-2946-6-9-S4.zip › bestMatchVisualizer/scripts/libs/ckeditor/plugins/onchange/docs/install.html]

OnChange event plugin


# On Change event Plugin for CKEditor

## Introduction

This is a plugin that tries to fire a 'change' event whenever the content of a CKEditor instance is changed.

### Author:

Alfonso Martínez de Lizarrondo

### Sponsored by:

Falcana

### Version history:

1. 1.0: 21-January-2011. First version.
2. 1.1: 3-September-2011. Fixed issues with the UndoManager events. Detect changes in Source mode.
3. 1.2: 18-September-2011. Avoid too many events in CKEditor 3.6.2. Filter keyboard to skip control and movement keys.
4. 1.3: 22-December-2011 Avoid firing the event after the editor has been destroyed.
5. 1.4: 7-September-2012 Don't fire events if the editor is readonly, thanks to Ulrich Gabor. Included code to use Mutation Observers.
6. 1.5: 20-October-2012 Detect Cut and Paste for IE in source mode thanks to Jacki.
7. 1.6: 18-November-2012 Detect multibyte characters thanks to Wouter.
8. 1.7: 6-December-2012 Compatibility with Source mode in CKEditor 4.
9. 1.8: 8-June-2013 Use setInterval fix by Roman Minkin.

## Installation

### 1. Copying the files

Extract the contents of the zip in you plugins directory, so it ends up like
this

```
ckeditor\
	...
	images\
	lang\
	plugins\
		...
		onchange\
			plugin.js
			docs\
				install.html
		...
	skins\
	themes\
```

### 2. Adding it to CKEditor

Now add the plugin in your *config.js* or custom js configuration
file:
`config.extraPlugins='onchange';`

### 3. Configuration

You can limit the minimum time between changes to avoid getting too many events fired:
`config.minimumChangeMilliseconds = 100; // 100 milliseconds (default value)` .

### 4. Use it

Write your listener for the new 'change' event and perform whatever action you need there.
`editor.on( 'change', function(e) { console.log(e) });` .

## Disclaimers

CKEditor is © CKSource.com
