# Supplementary material for: A new method for the comparison of 1H NMR predictors based on tree-similarity of spectra
Source: J Cheminform. 2014 Mar 25;6:9. doi: 10.1186/1758-2946-6-9 (PMC3987679; doi:10.1186/1758-2946-6-9)
Supplement: Additional file 4 — Directory containing a graphical tool to benchmark new predictions (submitted as input file in the correct format) with the predictions shown in this publication. The graphical tool provided in this compressed archive allows to compute and visualize the results for a new input file containing a new set of predictions. It consist in a web page (index.html) that can be accessed locally or remotely if the directory is placed on a server. The input file is simply drag and dropped on the webpage in order to start the computation of the complete similarity matrix and on the different statistical indicator including the curve of Figure 1. [file 1758-2946-6-9-S4.zip › bestMatchVisualizer/scripts/libs/ckeditor/samples/ajax.html]

Ajax — CKEditor Sample


# CKEditor Samples » Create and Destroy Editor Instances for Ajax Applications

This sample shows how to create and destroy CKEditor instances on the fly. After the removal of CKEditor the content created inside the editing
area will be displayed in a `<div>` element.

For details of how to create this setup check the source code of this sample page
for JavaScript code responsible for the creation and destruction of a CKEditor instance.

Click the buttons to create and remove a CKEditor instance.

Edited Contents:

---

CKEditor - The text editor for the Internet - http://ckeditor.com

Copyright © 2003-2013, CKSource - Frederico
Knabben. All rights reserved.
