# Supplementary material for: A new method for the comparison of 1H NMR predictors based on tree-similarity of spectra
Source: J Cheminform. 2014 Mar 25;6:9. doi: 10.1186/1758-2946-6-9 (PMC3987679; doi:10.1186/1758-2946-6-9)
Supplement: Additional file 4 — Directory containing a graphical tool to benchmark new predictions (submitted as input file in the correct format) with the predictions shown in this publication. The graphical tool provided in this compressed archive allows to compute and visualize the results for a new input file containing a new set of predictions. It consist in a web page (index.html) that can be accessed locally or remotely if the directory is placed on a server. The input file is simply drag and dropped on the webpage in order to start the computation of the complete similarity matrix and on the different statistical indicator including the curve of Figure 1. [file 1758-2946-6-9-S4.zip › bestMatchVisualizer/scripts/libs/ckeditor/samples/api.html]

API Usage — CKEditor Sample


# CKEditor Samples » Using CKEditor JavaScript API

This sample shows how to use the
CKEditor JavaScript API
to interact with the editor at runtime.

For details on how to create this setup check the source code of this sample page.

**CKEditor requires JavaScript to run**. In a browser with no JavaScript
support, like yours, you should still see the contents (HTML data) and you should
be able to edit it normally, without a rich editor interface.

<p>This is some <strong>sample text</strong>. You are using <a href="http://ckeditor.com/">CKEditor</a>.</p>

<h2>Test</h2><p>This is some <a href="/Test1.html">sample</a> HTML code.</p>
  
  

  
 First line with some leading whitespaces.
Second line of text preceded by two line breaks.

---

CKEditor - The text editor for the Internet - http://ckeditor.com

Copyright © 2003-2013, CKSource - Frederico
Knabben. All rights reserved.
