# Supplementary material for: A new method for the comparison of 1H NMR predictors based on tree-similarity of spectra
Source: J Cheminform. 2014 Mar 25;6:9. doi: 10.1186/1758-2946-6-9 (PMC3987679; doi:10.1186/1758-2946-6-9)
Supplement: Additional file 4 — Directory containing a graphical tool to benchmark new predictions (submitted as input file in the correct format) with the predictions shown in this publication. The graphical tool provided in this compressed archive allows to compute and visualize the results for a new input file containing a new set of predictions. It consist in a web page (index.html) that can be accessed locally or remotely if the directory is placed on a server. The input file is simply drag and dropped on the webpage in order to start the computation of the complete similarity matrix and on the different statistical indicator including the curve of Figure 1. [file 1758-2946-6-9-S4.zip › bestMatchVisualizer/scripts/libs/ckeditor/samples/appendto.html]

CKEDITOR.appendTo — CKEditor Sample


# CKEditor Samples » Append To Page Element Using JavaScript Code

`CKEDITOR.appendTo` is basically to place editors
inside existing DOM elements. Unlike `CKEDITOR.replace`,
a target container to be replaced is no longer necessary. A new editor
instance is inserted directly wherever it is desired.

```
CKEDITOR.appendTo( 'container_id',
	{ /* Configuration options to be used. */ }
	'Editor content to be used.'
);
```

  

---

CKEditor - The text editor for the Internet - http://ckeditor.com

Copyright © 2003-2013, CKSource - Frederico
Knabben. All rights reserved.
