# Supplementary material for: A new method for the comparison of 1H NMR predictors based on tree-similarity of spectra
Source: J Cheminform. 2014 Mar 25;6:9. doi: 10.1186/1758-2946-6-9 (PMC3987679; doi:10.1186/1758-2946-6-9)
Supplement: Additional file 4 — Directory containing a graphical tool to benchmark new predictions (submitted as input file in the correct format) with the predictions shown in this publication. The graphical tool provided in this compressed archive allows to compute and visualize the results for a new input file containing a new set of predictions. It consist in a web page (index.html) that can be accessed locally or remotely if the directory is placed on a server. The input file is simply drag and dropped on the webpage in order to start the computation of the complete similarity matrix and on the different statistical indicator including the curve of Figure 1. [file 1758-2946-6-9-S4.zip › bestMatchVisualizer/scripts/libs/ckeditor/samples/datafiltering.html]

Data Filtering — CKEditor Sample


# CKEditor Samples » Data Filtering and Features Activation

This sample page demonstrates the idea of Advanced Content Filter
(ACF), a sophisticated
tool that takes control over what kind of data is accepted by the editor and what
kind of output is produced.

## When and what is being filtered?

ACF controls
**every single source of data** that comes to the editor.
It process both HTML that is inserted manually (i.e. pasted by the user)
and programmatically like:

```
editor.setData( '<p>Hello world!</p>' );
```

ACF discards invalid,
useless HTML tags and attributes so the editor remains "clean" during
runtime. ACF behaviour
can be configured and adjusted for a particular case to prevent the
output HTML (i.e. in CMS systems) from being polluted.
This kind of filtering is a first, client-side line of defense
against "tag soups",
the tool that precisely restricts which tags, attributes and styles
are allowed (desired). When properly configured, ACF
is an easy and fast way to produce a high-quality, intentionally filtered HTML.

### How to configure or disable ACF?

Advanced Content Filter is enabled by default, working in "automatic mode", yet
it provides a set of easy rules that allow adjusting filtering rules
and disabling the entire feature when necessary. The config property
responsible for this feature is `config.allowedContent`.

By "automatic mode" is meant that loaded plugins decide which kind
of content is enabled and which is not. For example, if the link
plugin is loaded it implies that `<a>` tag is
automatically allowed. Each plugin is given a set
of predefined ACF rules
that control the editor until  `config.allowedContent`
is defined manually.

Let's assume our intention is to restrict the editor to accept (produce) **paragraphs
only: no attributes, no styles, no other tags**.
With ACF
this is very simple. Basically set  `config.allowedContent` to `'p'`:

```
var editor = CKEDITOR.replace( textarea_id, {
	allowedContent: 'p'
} );
```

Now try to play with allowed content:

```
// Trying to insert disallowed tag and attribute.
editor.setData( '<p style="color: red">Hello <em>world</em>!</p>' );
alert( editor.getData() );

// Filtered data is returned.
"<p>Hello world!</p>"
```

What happened? Since `config.allowedContent: 'p'` is set the editor assumes
that only plain `<p>` are accepted. Nothing more. This is why
`style` attribute and `<em>` tag are gone. The same
filtering would happen if we pasted disallowed HTML into this editor.

This is just a small sample of what ACF
can do. To know more, please refer to the sample section below and
the official Advanced Content Filter guide.

You may, of course, want CKEditor to avoid filtering of any kind.
To get rid of ACF,
basically set  `config.allowedContent` to `true` like this:

```
CKEDITOR.replace( textarea_id, {
	allowedContent: true
} );
```

## Beyond data flow: Features activation

ACF is far more than
I/O control: the entire
UI of the editor is adjusted to what
filters restrict. For example: if `<a>` tag is
**disallowed**
by ACF,
then accordingly `link` command, toolbar button and link dialog
are also disabled. Editor is smart: it knows which features must be
removed from the interface to match filtering rules.

CKEditor can be far more specific. If `<a>` tag is
**allowed** by filtering rules to be used but it is restricted
to have only one attribute (`href`)
`config.allowedContent = 'a[!href]'`, then
"Target" tab of the link dialog is automatically disabled as `target`
attribute isn't included in ACF rules
for `<a>`. This behaviour applies to dialog fields, context
menus and toolbar buttons.

## Sample configurations

There are several editor instances below that present different
ACF setups. **All of them,
except the last inline instance, share the same HTML content** to visualize
how different filtering rules affect the same input data.

Editor 1:

This editor is using default configuration ("automatic mode"). It means that
 `config.allowedContent` is defined by loaded plugins.
Each plugin extends filtering rules to make it's own associated content
available for the user.

<h1><img alt="Saturn V carrying Apollo 11" class="right" src="assets/sample.jpg"/> Apollo 11</h1> <p><b>Apollo 11</b> was the spaceflight that landed the first humans, Americans <a href="http://en.wikipedia.org/wiki/Neil\_Armstrong" title="Neil Armstrong">Neil Armstrong</a> and <a href="http://en.wikipedia.org/wiki/Buzz\_Aldrin" title="Buzz Aldrin">Buzz Aldrin</a>, on the Moon on July 20, 1969, at 20:18 UTC. Armstrong became the first to step onto the lunar surface 6 hours later on July 21 at 02:56 UTC.</p> <p>Armstrong spent about <s>three and a half</s> two and a half hours outside the spacecraft, Aldrin slightly less; and together they collected 47.5 pounds (21.5&nbsp;kg) of lunar material for return to Earth. A third member of the mission, <a href="http://en.wikipedia.org/wiki/Michael\_Collins\_(astronaut)" title="Michael Collins (astronaut)">Michael Collins</a>, piloted the <a href="http://en.wikipedia.org/wiki/Apollo\_Command/Service\_Module" title="Apollo Command/Service Module">command</a> spacecraft alone in lunar orbit until Armstrong and Aldrin returned to it for the trip back to Earth.</p> <h2>Broadcasting and <em>quotes</em> <a id="quotes" name="quotes"></a></h2> <p>Broadcast on live TV to a world-wide audience, Armstrong stepped onto the lunar surface and described the event as:</p> <blockquote><p>One small step for [a] man, one giant leap for mankind.</p></blockquote> <p>Apollo 11 effectively ended the <a href="http://en.wikipedia.org/wiki/Space\_Race" title="Space Race">Space Race</a> and fulfilled a national goal proposed in 1961 by the late U.S. President <a href="http://en.wikipedia.org/wiki/John\_F.\_Kennedy" title="John F. Kennedy">John F. Kennedy</a> in a speech before the United States Congress:</p> <blockquote><p>[...] before this decade is out, of landing a man on the Moon and returning him safely to the Earth.</p></blockquote> <h2>Technical details <a id="tech-details" name="tech-details"></a></h2> <table align="right" border="1" bordercolor="#ccc" cellpadding="5" cellspacing="0" style="border-collapse:collapse;margin:10px 0 10px 15px;"> <caption><strong>Mission crew</strong></caption> <thead> <tr> <th scope="col">Position</th> <th scope="col">Astronaut</th> </tr> </thead> <tbody> <tr> <td>Commander</td> <td>Neil A. Armstrong</td> </tr> <tr> <td>Command Module Pilot</td> <td>Michael Collins</td> </tr> <tr> <td>Lunar Module Pilot</td> <td>Edwin &quot;Buzz&quot; E. Aldrin, Jr.</td> </tr> </tbody> </table> <p>Launched by a <strong>Saturn V</strong> rocket from <a href="http://en.wikipedia.org/wiki/Kennedy\_Space\_Center" title="Kennedy Space Center">Kennedy Space Center</a> in Merritt Island, Florida on July 16, Apollo 11 was the fifth manned mission of <a href="http://en.wikipedia.org/wiki/NASA" title="NASA">NASA</a>&#39;s Apollo program. The Apollo spacecraft had three parts:</p> <ol> <li><strong>Command Module</strong> with a cabin for the three astronauts which was the only part which landed back on Earth</li> <li><strong>Service Module</strong> which supported the Command Module with propulsion, electrical power, oxygen and water</li> <li><strong>Lunar Module</strong> for landing on the Moon.</li> </ol> <p>After being sent to the Moon by the Saturn V&#39;s upper stage, the astronauts separated the spacecraft from it and travelled for three days until they entered into lunar orbit. Armstrong and Aldrin then moved into the Lunar Module and landed in the <a href="http://en.wikipedia.org/wiki/Mare\_Tranquillitatis" title="Mare Tranquillitatis">Sea of Tranquility</a>. They stayed a total of about 21 and a half hours on the lunar surface. After lifting off in the upper part of the Lunar Module and rejoining Collins in the Command Module, they returned to Earth and landed in the <a href="http://en.wikipedia.org/wiki/Pacific\_Ocean" title="Pacific Ocean">Pacific Ocean</a> on July 24.</p> <hr/> <p style="text-align: right;"><small>Source: <a href="http://en.wikipedia.org/wiki/Apollo\_11">Wikipedia.org</a></small></p>

  

Editor 2:

This editor is using a custom configuration for
ACF:

```
CKEDITOR.replace( 'editor2', {
	allowedContent:
		'h1 h2 h3 p blockquote strong em;' +
		'a[!href];' +
		'img(left,right)[!src,alt,width,height];' +
		'table tr th td caption;' +
		'span{!font-family};' +'
		'span{!color};' +
		'span(!marker);' +
		'del ins'
} );
```

The following rules may require additional explanation:

- `h1 h2 h3 p blockquote strong em` - These tags
  are accepted by the editor. Any tag attributes will be discarded.
- `a[!href]` - `href` attribute is obligatory
  for `<a>` tag. Tags without this attribute
  are disarded. No other attribute will be accepted.
- `img(left,right)[!src,alt,width,height]` - `src`
  attribute is obligatory for `<img>` tag.
  `alt`, `width`, `height`
  and `class` attributes are accepted but
  `class` must be either `class="left"`
  or `class="right"`
- `table tr th td caption` - These tags
  are accepted by the editor. Any tag attributes will be discarded.
- `span{!font-family}`, `span{!color}`,
  `span(!marker)` - `<span>` tags
  will be accepted if either `font-family` or
  `color` style is set or `class="marker"`
  is present.
- `del ins` - These tags
  are accepted by the editor. Any tag attributes will be discarded.

Please note that **UI of the
editor is different**. It's a response to what happened to the filters.
Since `text-align` isn't allowed, the align toolbar is gone.
The same thing happened to subscript/superscript, strike, underline
(`<u>`, `<sub>`, `<sup>`
are disallowed by  `config.allowedContent`) and many other buttons.

<h1><img alt="Saturn V carrying Apollo 11" class="right" src="assets/sample.jpg"/> Apollo 11</h1> <p><b>Apollo 11</b> was the spaceflight that landed the first humans, Americans <a href="http://en.wikipedia.org/wiki/Neil\_Armstrong" title="Neil Armstrong">Neil Armstrong</a> and <a href="http://en.wikipedia.org/wiki/Buzz\_Aldrin" title="Buzz Aldrin">Buzz Aldrin</a>, on the Moon on July 20, 1969, at 20:18 UTC. Armstrong became the first to step onto the lunar surface 6 hours later on July 21 at 02:56 UTC.</p> <p>Armstrong spent about <s>three and a half</s> two and a half hours outside the spacecraft, Aldrin slightly less; and together they collected 47.5 pounds (21.5&nbsp;kg) of lunar material for return to Earth. A third member of the mission, <a href="http://en.wikipedia.org/wiki/Michael\_Collins\_(astronaut)" title="Michael Collins (astronaut)">Michael Collins</a>, piloted the <a href="http://en.wikipedia.org/wiki/Apollo\_Command/Service\_Module" title="Apollo Command/Service Module">command</a> spacecraft alone in lunar orbit until Armstrong and Aldrin returned to it for the trip back to Earth.</p> <h2>Broadcasting and <em>quotes</em> <a id="quotes" name="quotes"></a></h2> <p>Broadcast on live TV to a world-wide audience, Armstrong stepped onto the lunar surface and described the event as:</p> <blockquote><p>One small step for [a] man, one giant leap for mankind.</p></blockquote> <p>Apollo 11 effectively ended the <a href="http://en.wikipedia.org/wiki/Space\_Race" title="Space Race">Space Race</a> and fulfilled a national goal proposed in 1961 by the late U.S. President <a href="http://en.wikipedia.org/wiki/John\_F.\_Kennedy" title="John F. Kennedy">John F. Kennedy</a> in a speech before the United States Congress:</p> <blockquote><p>[...] before this decade is out, of landing a man on the Moon and returning him safely to the Earth.</p></blockquote> <h2>Technical details <a id="tech-details" name="tech-details"></a></h2> <table align="right" border="1" bordercolor="#ccc" cellpadding="5" cellspacing="0" style="border-collapse:collapse;margin:10px 0 10px 15px;"> <caption><strong>Mission crew</strong></caption> <thead> <tr> <th scope="col">Position</th> <th scope="col">Astronaut</th> </tr> </thead> <tbody> <tr> <td>Commander</td> <td>Neil A. Armstrong</td> </tr> <tr> <td>Command Module Pilot</td> <td>Michael Collins</td> </tr> <tr> <td>Lunar Module Pilot</td> <td>Edwin &quot;Buzz&quot; E. Aldrin, Jr.</td> </tr> </tbody> </table> <p>Launched by a <strong>Saturn V</strong> rocket from <a href="http://en.wikipedia.org/wiki/Kennedy\_Space\_Center" title="Kennedy Space Center">Kennedy Space Center</a> in Merritt Island, Florida on July 16, Apollo 11 was the fifth manned mission of <a href="http://en.wikipedia.org/wiki/NASA" title="NASA">NASA</a>&#39;s Apollo program. The Apollo spacecraft had three parts:</p> <ol> <li><strong>Command Module</strong> with a cabin for the three astronauts which was the only part which landed back on Earth</li> <li><strong>Service Module</strong> which supported the Command Module with propulsion, electrical power, oxygen and water</li> <li><strong>Lunar Module</strong> for landing on the Moon.</li> </ol> <p>After being sent to the Moon by the Saturn V&#39;s upper stage, the astronauts separated the spacecraft from it and travelled for three days until they entered into lunar orbit. Armstrong and Aldrin then moved into the Lunar Module and landed in the <a href="http://en.wikipedia.org/wiki/Mare\_Tranquillitatis" title="Mare Tranquillitatis">Sea of Tranquility</a>. They stayed a total of about 21 and a half hours on the lunar surface. After lifting off in the upper part of the Lunar Module and rejoining Collins in the Command Module, they returned to Earth and landed in the <a href="http://en.wikipedia.org/wiki/Pacific\_Ocean" title="Pacific Ocean">Pacific Ocean</a> on July 24.</p> <hr/> <p style="text-align: right;"><small>Source: <a href="http://en.wikipedia.org/wiki/Apollo\_11">Wikipedia.org</a></small></p>

  

Editor 3:

This editor is using a custom configuration for
ACF.
Note that filters can be configured as an object literal
as an alternative to a string-based definition.

```
CKEDITOR.replace( 'editor3', {
	allowedContent: {
		'b i ul ol big small': true,
		'h1 h2 h3 p blockquote li': {
			styles: 'text-align'
		},
		a: { attributes: '!href,target' },
		img: {
			attributes: '!src,alt',
			styles: 'width,height',
			classes: 'left,right'
		}
	}
} );
```

<h1><img alt="Saturn V carrying Apollo 11" class="right" src="assets/sample.jpg"/> Apollo 11</h1> <p><b>Apollo 11</b> was the spaceflight that landed the first humans, Americans <a href="http://en.wikipedia.org/wiki/Neil\_Armstrong" title="Neil Armstrong">Neil Armstrong</a> and <a href="http://en.wikipedia.org/wiki/Buzz\_Aldrin" title="Buzz Aldrin">Buzz Aldrin</a>, on the Moon on July 20, 1969, at 20:18 UTC. Armstrong became the first to step onto the lunar surface 6 hours later on July 21 at 02:56 UTC.</p> <p>Armstrong spent about <s>three and a half</s> two and a half hours outside the spacecraft, Aldrin slightly less; and together they collected 47.5 pounds (21.5&nbsp;kg) of lunar material for return to Earth. A third member of the mission, <a href="http://en.wikipedia.org/wiki/Michael\_Collins\_(astronaut)" title="Michael Collins (astronaut)">Michael Collins</a>, piloted the <a href="http://en.wikipedia.org/wiki/Apollo\_Command/Service\_Module" title="Apollo Command/Service Module">command</a> spacecraft alone in lunar orbit until Armstrong and Aldrin returned to it for the trip back to Earth.</p> <h2>Broadcasting and <em>quotes</em> <a id="quotes" name="quotes"></a></h2> <p>Broadcast on live TV to a world-wide audience, Armstrong stepped onto the lunar surface and described the event as:</p> <blockquote><p>One small step for [a] man, one giant leap for mankind.</p></blockquote> <p>Apollo 11 effectively ended the <a href="http://en.wikipedia.org/wiki/Space\_Race" title="Space Race">Space Race</a> and fulfilled a national goal proposed in 1961 by the late U.S. President <a href="http://en.wikipedia.org/wiki/John\_F.\_Kennedy" title="John F. Kennedy">John F. Kennedy</a> in a speech before the United States Congress:</p> <blockquote><p>[...] before this decade is out, of landing a man on the Moon and returning him safely to the Earth.</p></blockquote> <h2>Technical details <a id="tech-details" name="tech-details"></a></h2> <table align="right" border="1" bordercolor="#ccc" cellpadding="5" cellspacing="0" style="border-collapse:collapse;margin:10px 0 10px 15px;"> <caption><strong>Mission crew</strong></caption> <thead> <tr> <th scope="col">Position</th> <th scope="col">Astronaut</th> </tr> </thead> <tbody> <tr> <td>Commander</td> <td>Neil A. Armstrong</td> </tr> <tr> <td>Command Module Pilot</td> <td>Michael Collins</td> </tr> <tr> <td>Lunar Module Pilot</td> <td>Edwin &quot;Buzz&quot; E. Aldrin, Jr.</td> </tr> </tbody> </table> <p>Launched by a <strong>Saturn V</strong> rocket from <a href="http://en.wikipedia.org/wiki/Kennedy\_Space\_Center" title="Kennedy Space Center">Kennedy Space Center</a> in Merritt Island, Florida on July 16, Apollo 11 was the fifth manned mission of <a href="http://en.wikipedia.org/wiki/NASA" title="NASA">NASA</a>&#39;s Apollo program. The Apollo spacecraft had three parts:</p> <ol> <li><strong>Command Module</strong> with a cabin for the three astronauts which was the only part which landed back on Earth</li> <li><strong>Service Module</strong> which supported the Command Module with propulsion, electrical power, oxygen and water</li> <li><strong>Lunar Module</strong> for landing on the Moon.</li> </ol> <p>After being sent to the Moon by the Saturn V&#39;s upper stage, the astronauts separated the spacecraft from it and travelled for three days until they entered into lunar orbit. Armstrong and Aldrin then moved into the Lunar Module and landed in the <a href="http://en.wikipedia.org/wiki/Mare\_Tranquillitatis" title="Mare Tranquillitatis">Sea of Tranquility</a>. They stayed a total of about 21 and a half hours on the lunar surface. After lifting off in the upper part of the Lunar Module and rejoining Collins in the Command Module, they returned to Earth and landed in the <a href="http://en.wikipedia.org/wiki/Pacific\_Ocean" title="Pacific Ocean">Pacific Ocean</a> on July 24.</p> <hr/> <p style="text-align: right;"><small>Source: <a href="http://en.wikipedia.org/wiki/Apollo\_11">Wikipedia.org</a></small></p>

  

Editor 4:

This editor is using a custom set of plugins and buttons.

```
CKEDITOR.replace( 'editor4', {
	removePlugins: 'bidi,font,forms,flash,horizontalrule,iframe,justify,table,tabletools,smiley',
	removeButtons: 'Anchor,Underline,Strike,Subscript,Superscript,Image',
	format_tags: 'p;h1;h2;h3;pre;address'
} );
```

As you can see, removing plugins and buttons implies filtering.
Several tags are not allowed in the editor because there's no
plugin/button that is responsible for creating and editing this
kind of content (for example: the image is missing because
of `removeButtons: 'Image'`). The conclusion is that
ACF works "backwards"
as well: **modifying UI
elements is changing allowed content rules**.

<h1><img alt="Saturn V carrying Apollo 11" class="right" src="assets/sample.jpg"/> Apollo 11</h1> <p><b>Apollo 11</b> was the spaceflight that landed the first humans, Americans <a href="http://en.wikipedia.org/wiki/Neil\_Armstrong" title="Neil Armstrong">Neil Armstrong</a> and <a href="http://en.wikipedia.org/wiki/Buzz\_Aldrin" title="Buzz Aldrin">Buzz Aldrin</a>, on the Moon on July 20, 1969, at 20:18 UTC. Armstrong became the first to step onto the lunar surface 6 hours later on July 21 at 02:56 UTC.</p> <p>Armstrong spent about <s>three and a half</s> two and a half hours outside the spacecraft, Aldrin slightly less; and together they collected 47.5 pounds (21.5&nbsp;kg) of lunar material for return to Earth. A third member of the mission, <a href="http://en.wikipedia.org/wiki/Michael\_Collins\_(astronaut)" title="Michael Collins (astronaut)">Michael Collins</a>, piloted the <a href="http://en.wikipedia.org/wiki/Apollo\_Command/Service\_Module" title="Apollo Command/Service Module">command</a> spacecraft alone in lunar orbit until Armstrong and Aldrin returned to it for the trip back to Earth.</p> <h2>Broadcasting and <em>quotes</em> <a id="quotes" name="quotes"></a></h2> <p>Broadcast on live TV to a world-wide audience, Armstrong stepped onto the lunar surface and described the event as:</p> <blockquote><p>One small step for [a] man, one giant leap for mankind.</p></blockquote> <p>Apollo 11 effectively ended the <a href="http://en.wikipedia.org/wiki/Space\_Race" title="Space Race">Space Race</a> and fulfilled a national goal proposed in 1961 by the late U.S. President <a href="http://en.wikipedia.org/wiki/John\_F.\_Kennedy" title="John F. Kennedy">John F. Kennedy</a> in a speech before the United States Congress:</p> <blockquote><p>[...] before this decade is out, of landing a man on the Moon and returning him safely to the Earth.</p></blockquote> <h2>Technical details <a id="tech-details" name="tech-details"></a></h2> <table align="right" border="1" bordercolor="#ccc" cellpadding="5" cellspacing="0" style="border-collapse:collapse;margin:10px 0 10px 15px;"> <caption><strong>Mission crew</strong></caption> <thead> <tr> <th scope="col">Position</th> <th scope="col">Astronaut</th> </tr> </thead> <tbody> <tr> <td>Commander</td> <td>Neil A. Armstrong</td> </tr> <tr> <td>Command Module Pilot</td> <td>Michael Collins</td> </tr> <tr> <td>Lunar Module Pilot</td> <td>Edwin &quot;Buzz&quot; E. Aldrin, Jr.</td> </tr> </tbody> </table> <p>Launched by a <strong>Saturn V</strong> rocket from <a href="http://en.wikipedia.org/wiki/Kennedy\_Space\_Center" title="Kennedy Space Center">Kennedy Space Center</a> in Merritt Island, Florida on July 16, Apollo 11 was the fifth manned mission of <a href="http://en.wikipedia.org/wiki/NASA" title="NASA">NASA</a>&#39;s Apollo program. The Apollo spacecraft had three parts:</p> <ol> <li><strong>Command Module</strong> with a cabin for the three astronauts which was the only part which landed back on Earth</li> <li><strong>Service Module</strong> which supported the Command Module with propulsion, electrical power, oxygen and water</li> <li><strong>Lunar Module</strong> for landing on the Moon.</li> </ol> <p>After being sent to the Moon by the Saturn V&#39;s upper stage, the astronauts separated the spacecraft from it and travelled for three days until they entered into lunar orbit. Armstrong and Aldrin then moved into the Lunar Module and landed in the <a href="http://en.wikipedia.org/wiki/Mare\_Tranquillitatis" title="Mare Tranquillitatis">Sea of Tranquility</a>. They stayed a total of about 21 and a half hours on the lunar surface. After lifting off in the upper part of the Lunar Module and rejoining Collins in the Command Module, they returned to Earth and landed in the <a href="http://en.wikipedia.org/wiki/Pacific\_Ocean" title="Pacific Ocean">Pacific Ocean</a> on July 24.</p> <hr/> <p style="text-align: right;"><small>Source: <a href="http://en.wikipedia.org/wiki/Apollo\_11">Wikipedia.org</a></small></p>

  

Editor 5:

This editor is built on editable `<h1>` element.
ACF takes care of
what can be included in `<h1>`. Note that there
are no block styles in Styles combo. Also why lists, indentation,
blockquote, div, form and other buttons are missing.

ACF makes sure that
no disallowed tags will come to `<h1>` so the final
markup is valid. If the user tried to paste some invalid HTML
into this editor (let's say a list), it would be automatically
converted into plain text.

# *Apollo 11* was the spaceflight that landed the first humans, Americans Neil Armstrong and Buzz Aldrin, on the Moon on July 20, 1969, at 20:18 UTC.

---

CKEditor - The text editor for the Internet - http://ckeditor.com

Copyright © 2003-2013, CKSource - Frederico
Knabben. All rights reserved.
