# Supplementary material for: A new method for the comparison of 1H NMR predictors based on tree-similarity of spectra
Source: J Cheminform. 2014 Mar 25;6:9. doi: 10.1186/1758-2946-6-9 (PMC3987679; doi:10.1186/1758-2946-6-9)
Supplement: Additional file 4 — Directory containing a graphical tool to benchmark new predictions (submitted as input file in the correct format) with the predictions shown in this publication. The graphical tool provided in this compressed archive allows to compute and visualize the results for a new input file containing a new set of predictions. It consist in a web page (index.html) that can be accessed locally or remotely if the directory is placed on a server. The input file is simply drag and dropped on the webpage in order to start the computation of the complete similarity matrix and on the different statistical indicator including the curve of Figure 1. [file 1758-2946-6-9-S4.zip › bestMatchVisualizer/scripts/libs/ckeditor/samples/divreplace.html]

Replace DIV — CKEditor Sample


# CKEditor Samples » Replace DIV with CKEditor on the Fly

This sample shows how to automatically replace `<div>` elements
with a CKEditor instance on the fly, following user's doubleclick. The content
that was previously placed inside the `<div>` element will now
be moved into CKEditor editing area.

For details on how to create this setup check the source code of this sample page.

Double-click any of the following `<div>` elements to transform them into
editor instances.

### Part 1

Lorem ipsum dolor sit amet, consectetuer adipiscing elit. Cras et ipsum quis mi
semper accumsan. Integer pretium dui id massa. Suspendisse in nisl sit amet urna
rutrum imperdiet. Nulla eu tellus. Donec ante nisi, ullamcorper quis, fringilla
nec, sagittis eleifend, pede. Nulla commodo interdum massa. Donec id metus. Fusce
eu ipsum. Suspendisse auctor. Phasellus fermentum porttitor risus.

### Part 2

Lorem ipsum dolor sit amet, consectetuer adipiscing elit. Cras et ipsum quis mi
semper accumsan. Integer pretium dui id massa. Suspendisse in nisl sit amet urna
rutrum imperdiet. Nulla eu tellus. Donec ante nisi, ullamcorper quis, fringilla
nec, sagittis eleifend, pede. Nulla commodo interdum massa. Donec id metus. Fusce
eu ipsum. Suspendisse auctor. Phasellus fermentum porttitor risus.

Donec velit. Mauris massa. Vestibulum non nulla. Nam suscipit arcu nec elit. Phasellus
sollicitudin iaculis ante. Ut non mauris et sapien tincidunt adipiscing. Vestibulum
vitae leo. Suspendisse nec mi tristique nulla laoreet vulputate.

### Part 3

Lorem ipsum dolor sit amet, consectetuer adipiscing elit. Cras et ipsum quis mi
semper accumsan. Integer pretium dui id massa. Suspendisse in nisl sit amet urna
rutrum imperdiet. Nulla eu tellus. Donec ante nisi, ullamcorper quis, fringilla
nec, sagittis eleifend, pede. Nulla commodo interdum massa. Donec id metus. Fusce
eu ipsum. Suspendisse auctor. Phasellus fermentum porttitor risus.

---

CKEditor - The text editor for the Internet - http://ckeditor.com

Copyright © 2003-2013, CKSource - Frederico
Knabben. All rights reserved.
