# Supplementary material for: A new method for the comparison of 1H NMR predictors based on tree-similarity of spectra
Source: J Cheminform. 2014 Mar 25;6:9. doi: 10.1186/1758-2946-6-9 (PMC3987679; doi:10.1186/1758-2946-6-9)
Supplement: Additional file 4 — Directory containing a graphical tool to benchmark new predictions (submitted as input file in the correct format) with the predictions shown in this publication. The graphical tool provided in this compressed archive allows to compute and visualize the results for a new input file containing a new set of predictions. It consist in a web page (index.html) that can be accessed locally or remotely if the directory is placed on a server. The input file is simply drag and dropped on the webpage in order to start the computation of the complete similarity matrix and on the different statistical indicator including the curve of Figure 1. [file 1758-2946-6-9-S4.zip › bestMatchVisualizer/scripts/libs/ckeditor/samples/index.html]

CKEditor Samples


# CKEditor Samples

## Basic Samples

Replace textarea elements by class name
:   Automatic replacement of all textarea elements of a given class with a CKEditor instance.

Replace textarea elements by code
:   Replacement of textarea elements with CKEditor instances by using a JavaScript call.

## Basic Customization

User Interface color
:   Changing CKEditor User Interface color and adding a toolbar button that lets the user set the UI color.

User Interface languages
:   Changing CKEditor User Interface language and adding a drop-down list that lets the user choose the UI language.

## Plugins

Magicline pluginNew!
:   Using the Magicline plugin to access difficult focus spaces.

Full page support
:   CKEditor inserted with a JavaScript call and used to edit the whole page from <html> to </html>.

## Inline Editing New!

Massive inline editor creation New!
:   Turn all elements with `contentEditable = true` attribute into inline editors.

Convert element into an inline editor by code New!
:   Conversion of DOM elements into inline CKEditor instances by using a JavaScript call.

## Advanced Samples

Data filtering and features activation New!
:   Data filtering and automatic features activation basing on configuration.

Replace DIV elements on the fly
:   Transforming a `div` element into an instance of CKEditor with a mouse click.

Append editor instances
:   Appending editor instances to existing DOM elements.

Create and destroy editor instances for Ajax applications
:   Creating and destroying CKEditor instances on the fly and saving the contents entered into the editor window.

Basic usage of the API
:   Using the CKEditor JavaScript API to interact with the editor at runtime.

XHTML-compliant style
:   Configuring CKEditor to produce XHTML 1.1 compliant attributes and styles.

Read-only mode
:   Using the readOnly API to block introducing changes to the editor contents.

"Tab" key-based navigation New!
:   Navigating among editor instances with tab key.

Using the JavaScript API to customize dialog windows
:   Using the dialog windows API to customize dialog windows without changing the original editor code.

Using the "Enter" key in CKEditor
:   Configuring the behavior of *Enter* and *Shift+Enter* keys.

Output for Flash
:   Configuring CKEditor to produce HTML code that can be used with Adobe Flash.

Output HTML
:   Configuring CKEditor to produce legacy HTML 4 code.

Toolbar ConfigurationsNew!
:   Configuring CKEditor to display full or custom toolbar layout.

---

CKEditor - The text editor for the Internet - http://ckeditor.com

Copyright © 2003-2013, CKSource - Frederico Knabben. All rights reserved.
