# Supplementary material for: A new method for the comparison of 1H NMR predictors based on tree-similarity of spectra
Source: J Cheminform. 2014 Mar 25;6:9. doi: 10.1186/1758-2946-6-9 (PMC3987679; doi:10.1186/1758-2946-6-9)
Supplement: Additional file 4 — Directory containing a graphical tool to benchmark new predictions (submitted as input file in the correct format) with the predictions shown in this publication. The graphical tool provided in this compressed archive allows to compute and visualize the results for a new input file containing a new set of predictions. It consist in a web page (index.html) that can be accessed locally or remotely if the directory is placed on a server. The input file is simply drag and dropped on the webpage in order to start the computation of the complete similarity matrix and on the different statistical indicator including the curve of Figure 1. [file 1758-2946-6-9-S4.zip › bestMatchVisualizer/scripts/libs/ckeditor/samples/inlineall.html]

Massive inline editing — CKEditor Sample


# CKEditor Samples » Massive inline editing

This sample page demonstrates the inline editing feature - CKEditor instances will be created automatically from page elements with **contentEditable** attribute set to value **true**:

```
<div contenteditable="true" > ... </div>
```

Click inside of any element below to start editing.

## CKEditor Goes Inline!

### Lorem ipsum dolor sit amet dolor duis blandit vestibulum faucibus a, tortor.

Lorem ipsum dolor sit amet enim. Etiam ullamcorper. Suspendisse a pellentesque dui, non felis. Maecenas malesuada elit lectus felis, malesuada ultricies.

Curabitur et ligula. Ut molestie a, ultricies porta urna. Vestibulum commodo volutpat a, convallis ac, laoreet enim. Phasellus fermentum in, dolor. Pellentesque facilisis. Nulla imperdiet sit amet magna. Vestibulum dapibus, mauris nec malesuada fames ac.

### Fusce vitae porttitor

**Lorem ipsum dolor sit amet dolor. Duis blandit vestibulum faucibus a, tortor.**

Proin nunc justo felis mollis tincidunt, risus risus pede, posuere cubilia Curae, Nullam euismod, enim. Etiam nibh ultricies dolor ac dignissim erat volutpat. Vivamus fermentum nisl nulla sem in metus. Maecenas wisi. Donec nec erat volutpat.

> Fusce vitae porttitor a, euismod convallis nisl, blandit risus tortor, pretium.
> Vehicula vitae, imperdiet vel, ornare enim vel sodales rutrum

> Libero nunc, rhoncus ante ipsum non ipsum. Nunc eleifend pede turpis id sollicitudin fringilla. Phasellus ultrices, velit ac arcu.

Pellentesque nunc. Donec suscipit erat. Pellentesque habitant morbi tristique ullamcorper.

~~Mauris mattis feugiat lectus nec mauris. Nullam vitae ante.~~

### Integer condimentum sit amet

**Aenean nonummy a, mattis varius. Cras aliquet.**
Praesent magna non mattis ac, rhoncus nunc, rhoncus eget, cursus pulvinar mollis.

Proin id nibh. Sed eu libero posuere sed, lectus. Phasellus dui gravida gravida feugiat mattis ac, felis.

Integer condimentum sit amet, tempor elit odio, a dolor non ante at sapien. Sed ac lectus. Nulla ligula quis eleifend mi, id leo velit pede cursus arcu id nulla ac lectus. Phasellus vestibulum. Nunc viverra enim quis diam.

### Praesent wisi accumsan sit amet nibh

Donec ullamcorper, risus tortor, pretium porttitor. Morbi quam quis lectus non leo.

Integer faucibus scelerisque. Proin faucibus at, aliquet vulputate, odio at eros. Fusce gravida, erat vitae augue. Fusce urna fringilla gravida.

In hac habitasse platea dictumst. Praesent wisi accumsan sit amet nibh. Maecenas orci luctus a, lacinia quam sem, posuere commodo, odio condimentum tempor, pede semper risus. Suspendisse pede. In hac habitasse platea dictumst. Nam sed laoreet sit amet erat. Integer.

Quisque justo neque, mattis sed, fermentum ultrices **posuere cubilia Curae**, Vestibulum elit metus, quis placerat ut, lectus. Ut sagittis, nunc libero, egestas consequat lobortis velit rutrum ut, faucibus turpis. Fusce porttitor, nulla quis turpis. Nullam laoreet vel, consectetuer tellus suscipit ultricies, hendrerit wisi. Donec odio nec velit ac nunc sit amet, accumsan cursus aliquet. Vestibulum ante sit amet sagittis mi.

### Nullam laoreet vel consectetuer tellus suscipit

- Ut sagittis, nunc libero, egestas consequat lobortis velit rutrum ut, faucibus turpis.
- Fusce porttitor, nulla quis turpis. Nullam laoreet vel, consectetuer tellus suscipit ultricies, hendrerit wisi.
- Mauris eget tellus. Donec non felis. Nam eget dolor. Vestibulum enim. Donec.

Quisque justo neque, mattis sed, fermentum ultrices posuere cubilia Curae, Vestibulum elit metus, quis placerat ut, lectus.

Nullam laoreet vel, consectetuer tellus suscipit ultricies, hendrerit wisi. Ut sagittis, nunc libero, egestas consequat lobortis velit rutrum ut, faucibus turpis. Fusce porttitor, nulla quis turpis.

Donec odio nec velit ac nunc sit amet, accumsan cursus aliquet. Vestibulum ante sit amet sagittis mi. Sed in nonummy faucibus turpis. Mauris eget tellus. Donec non felis. Nam eget dolor. Vestibulum enim. Donec.

Tags of this article:

inline, editing, floating, CKEditor

---

CKEditor - The text editor for the Internet - 
http://ckeditor.com

Copyright © 2003-2013, CKSource
- Frederico Knabben. All rights reserved.
