# Supplementary material for: A new method for the comparison of 1H NMR predictors based on tree-similarity of spectra
Source: J Cheminform. 2014 Mar 25;6:9. doi: 10.1186/1758-2946-6-9 (PMC3987679; doi:10.1186/1758-2946-6-9)
Supplement: Additional file 4 — Directory containing a graphical tool to benchmark new predictions (submitted as input file in the correct format) with the predictions shown in this publication. The graphical tool provided in this compressed archive allows to compute and visualize the results for a new input file containing a new set of predictions. It consist in a web page (index.html) that can be accessed locally or remotely if the directory is placed on a server. The input file is simply drag and dropped on the webpage in order to start the computation of the complete similarity matrix and on the different statistical indicator including the curve of Figure 1. [file 1758-2946-6-9-S4.zip › bestMatchVisualizer/scripts/libs/ckeditor/samples/inlinebycode.html]

Inline Editing by Code — CKEditor Sample


# CKEditor Samples » Inline Editing by Code

This sample shows how to create an inline editor instance of CKEditor. It is created
with a JavaScript call using the following code:

```
// This property tells CKEditor to not activate every element with contenteditable=true element.
CKEDITOR.disableAutoInline = true;

var editor = CKEDITOR.inline( document.getElementById( 'editable' ) );
```

Note that `editable` in the code above is the `id`
attribute of the `<div>` element to be converted into an inline instance.

# Apollo 11

**Apollo 11** was the spaceflight that landed the first humans, Americans Neil Armstrong and Buzz Aldrin, on the Moon on July 20, 1969, at 20:18 UTC. Armstrong became the first to step onto the lunar surface 6 hours later on July 21 at 02:56 UTC.

Armstrong spent about ~~three and a half~~ two and a half hours outside the spacecraft, Aldrin slightly less; and together they collected 47.5 pounds (21.5 kg) of lunar material for return to Earth. A third member of the mission, Michael Collins, piloted the command spacecraft alone in lunar orbit until Armstrong and Aldrin returned to it for the trip back to Earth.

## Broadcasting and *quotes*

Broadcast on live TV to a world-wide audience, Armstrong stepped onto the lunar surface and described the event as:

> One small step for [a] man, one giant leap for mankind.

Apollo 11 effectively ended the Space Race and fulfilled a national goal proposed in 1961 by the late U.S. President John F. Kennedy in a speech before the United States Congress:

> [...] before this decade is out, of landing a man on the Moon and returning him safely to the Earth.

## Technical details

**Mission crew**

| Position | Astronaut |
| --- | --- |
| Commander | Neil A. Armstrong |
| Command Module Pilot | Michael Collins |
| Lunar Module Pilot | Edwin "Buzz" E. Aldrin, Jr. |

Launched by a **Saturn V** rocket from Kennedy Space Center in Merritt Island, Florida on July 16, Apollo 11 was the fifth manned mission of NASA's Apollo program. The Apollo spacecraft had three parts:

1. **Command Module** with a cabin for the three astronauts which was the only part which landed back on Earth
2. **Service Module** which supported the Command Module with propulsion, electrical power, oxygen and water
3. **Lunar Module** for landing on the Moon.

After being sent to the Moon by the Saturn V's upper stage, the astronauts separated the spacecraft from it and travelled for three days until they entered into lunar orbit. Armstrong and Aldrin then moved into the Lunar Module and landed in the Sea of Tranquility. They stayed a total of about 21 and a half hours on the lunar surface. After lifting off in the upper part of the Lunar Module and rejoining Collins in the Command Module, they returned to Earth and landed in the Pacific Ocean on July 24.

---

Source: Wikipedia.org

---

CKEditor - The text editor for the Internet - 
http://ckeditor.com

Copyright © 2003-2013, CKSource
- Frederico Knabben. All rights reserved.
