# Supplementary material for: A new method for the comparison of 1H NMR predictors based on tree-similarity of spectra
Source: J Cheminform. 2014 Mar 25;6:9. doi: 10.1186/1758-2946-6-9 (PMC3987679; doi:10.1186/1758-2946-6-9)
Supplement: Additional file 4 — Directory containing a graphical tool to benchmark new predictions (submitted as input file in the correct format) with the predictions shown in this publication. The graphical tool provided in this compressed archive allows to compute and visualize the results for a new input file containing a new set of predictions. It consist in a web page (index.html) that can be accessed locally or remotely if the directory is placed on a server. The input file is simply drag and dropped on the webpage in order to start the computation of the complete similarity matrix and on the different statistical indicator including the curve of Figure 1. [file 1758-2946-6-9-S4.zip › bestMatchVisualizer/scripts/libs/ckeditor/samples/plugins/dialog/dialog.html]

Using API to Customize Dialog Windows — CKEditor Sample


# CKEditor Samples » Using CKEditor Dialog API

This sample shows how to use the
CKEditor Dialog API
to customize CKEditor dialog windows without changing the original editor code.
The following customizations are being done in the example below:

For details on how to create this setup check the source code of this sample page.

A custom dialog is added to the editors using the `pluginsLoaded` event, from an external dialog definition file:

1. **Creating a custom dialog window** – "My Dialog" dialog window opened with the "My Dialog" toolbar button.
2. **Creating a custom button** – Add button to open the dialog with "My Dialog" toolbar button.

<p>This is some <strong>sample text</strong>. You are using <a href="http://ckeditor.com/">CKEditor</a>.</p>

The below editor modify the dialog definition of the above added dialog using the `dialogDefinition` event:

1. **Adding dialog tab** – Add new tab "My Tab" to dialog window.
2. **Removing a dialog window tab** – Remove "Second Tab" page from the dialog window.
3. **Adding dialog window fields** – Add "My Custom Field" to the dialog window.
4. **Removing dialog window field** – Remove "Select Field" selection field from the dialog window.
5. **Setting default values for dialog window fields** – Set default value of "Text Field" text field.
6. **Setup initial focus for dialog window** – Put initial focus on "My Custom Field" text field.

<p>This is some <strong>sample text</strong>. You are using <a href="http://ckeditor.com/">CKEditor</a>.</p>

---

CKEditor - The text editor for the Internet - http://ckeditor.com

Copyright © 2003-2013, CKSource - Frederico
Knabben. All rights reserved.
