# Supplementary material for: A new method for the comparison of 1H NMR predictors based on tree-similarity of spectra
Source: J Cheminform. 2014 Mar 25;6:9. doi: 10.1186/1758-2946-6-9 (PMC3987679; doi:10.1186/1758-2946-6-9)
Supplement: Additional file 4 — Directory containing a graphical tool to benchmark new predictions (submitted as input file in the correct format) with the predictions shown in this publication. The graphical tool provided in this compressed archive allows to compute and visualize the results for a new input file containing a new set of predictions. It consist in a web page (index.html) that can be accessed locally or remotely if the directory is placed on a server. The input file is simply drag and dropped on the webpage in order to start the computation of the complete similarity matrix and on the different statistical indicator including the curve of Figure 1. [file 1758-2946-6-9-S4.zip › bestMatchVisualizer/scripts/libs/ckeditor/samples/plugins/enterkey/enterkey.html]

ENTER Key Configuration — CKEditor Sample


# CKEditor Samples » ENTER Key Configuration

This sample shows how to configure the *Enter* and *Shift+Enter* keys
to perform actions specified in the
`enterMode`
and `shiftEnterMode`
parameters, respectively.
You can choose from the following options:

- **`ENTER_P`** – new `<p>` paragraphs are created;
- **`ENTER_BR`** – lines are broken with `<br>` elements;
- **`ENTER_DIV`** – new `<div>` blocks are created.

The sample code below shows how to configure CKEditor to create a `<div>` block when *Enter* key is pressed.

```
CKEDITOR.replace( 'textarea_id', {
	enterMode: CKEDITOR.ENTER_DIV
});
```

Note that `textarea_id` in the code above is the `id` attribute of
the `<textarea>` element to be replaced.

When *Enter* is pressed:  

Create a new <P> (recommended)
Create a new <DIV>
Break the line with a <BR>

When *Shift+Enter* is pressed:  

Create a new <P>
Create a new <DIV>
Break the line with a <BR> (recommended)

  


This is some <strong>sample text</strong>. You are using <a href="http://ckeditor.com/">CKEditor</a>.

---

CKEditor - The text editor for the Internet - http://ckeditor.com

Copyright © 2003-2013, CKSource - Frederico
Knabben. All rights reserved.
