# Supplementary material for: A new method for the comparison of 1H NMR predictors based on tree-similarity of spectra
Source: J Cheminform. 2014 Mar 25;6:9. doi: 10.1186/1758-2946-6-9 (PMC3987679; doi:10.1186/1758-2946-6-9)
Supplement: Additional file 4 — Directory containing a graphical tool to benchmark new predictions (submitted as input file in the correct format) with the predictions shown in this publication. The graphical tool provided in this compressed archive allows to compute and visualize the results for a new input file containing a new set of predictions. It consist in a web page (index.html) that can be accessed locally or remotely if the directory is placed on a server. The input file is simply drag and dropped on the webpage in order to start the computation of the complete similarity matrix and on the different statistical indicator including the curve of Figure 1. [file 1758-2946-6-9-S4.zip › bestMatchVisualizer/scripts/libs/ckeditor/samples/plugins/htmlwriter/outputforflash.html]

Output for Flash — CKEditor Sample


# CKEditor Samples » Producing Flash Compliant HTML Output

This sample shows how to configure CKEditor to output
HTML code that can be used with
Adobe Flash.
The code will contain a subset of standard HTML elements like `<b>`,
`<i>`, and `<p>` as well as HTML attributes.

To add a CKEditor instance outputting Flash compliant HTML code, load the editor using a standard
JavaScript call, and define CKEditor features to use HTML elements and attributes.

For details on how to create this setup check the source code of this sample page.

To see how it works, create some content in the editing area of CKEditor on the left
and send it to the Flash object on the right side of the page by using the
**Send to Flash** button.

|  |  |
| --- | --- |
| <p><b><font size="18" style="font-size:18px;">Flash and HTML</font></b></p><p>&nbsp;</p><p>It is possible to have <a href="http://ckeditor.com">CKEditor</a> creating content that will be later loaded inside <b>Flash</b> objects and animations.</p><p>&nbsp;</p><p>Flash has a few limitations when dealing with HTML:</p><p>&nbsp;</p><ul><li>It has limited support on tags.</li><li>There is no margin between block elements, like paragraphs.</li></ul> |  |

---

CKEditor - The text editor for the Internet - http://ckeditor.com

Copyright © 2003-2013, CKSource - Frederico
Knabben. All rights reserved.
