# Supplementary material for: A new method for the comparison of 1H NMR predictors based on tree-similarity of spectra
Source: J Cheminform. 2014 Mar 25;6:9. doi: 10.1186/1758-2946-6-9 (PMC3987679; doi:10.1186/1758-2946-6-9)
Supplement: Additional file 4 — Directory containing a graphical tool to benchmark new predictions (submitted as input file in the correct format) with the predictions shown in this publication. The graphical tool provided in this compressed archive allows to compute and visualize the results for a new input file containing a new set of predictions. It consist in a web page (index.html) that can be accessed locally or remotely if the directory is placed on a server. The input file is simply drag and dropped on the webpage in order to start the computation of the complete similarity matrix and on the different statistical indicator including the curve of Figure 1. [file 1758-2946-6-9-S4.zip › bestMatchVisualizer/scripts/libs/ckeditor/samples/plugins/htmlwriter/outputhtml.html]

HTML Compliant Output — CKEditor Sample


# CKEditor Samples » Producing HTML Compliant Output

This sample shows how to configure CKEditor to output valid
HTML 4.01 code.
Traditional HTML elements like `<b>`,
`<i>`, and `<font>` are used in place of
`<strong>`, `<em>`, and CSS styles.

To add a CKEditor instance outputting legacy HTML 4.01 code, load the editor using a standard
JavaScript call, and define CKEditor features to use the HTML compliant elements and attributes.

A snippet of the configuration code can be seen below; check the source of this page for
full definition:

```
CKEDITOR.replace( 'textarea_id', {
	coreStyles_bold: { element: 'b' },
	coreStyles_italic: { element: 'i' },

	fontSize_style: {
		element: 'font',
		attributes: { 'size': '#(size)' }
	}

	...
});
```

Editor 1:
<p>This is some <b>sample text</b>. You are using <a href="http://ckeditor.com/">CKEditor</a>.</p>

---

CKEditor - The text editor for the Internet - http://ckeditor.com

Copyright © 2003-2013, CKSource - Frederico
Knabben. All rights reserved.
