# Supplementary material for: A new method for the comparison of 1H NMR predictors based on tree-similarity of spectra
Source: J Cheminform. 2014 Mar 25;6:9. doi: 10.1186/1758-2946-6-9 (PMC3987679; doi:10.1186/1758-2946-6-9)
Supplement: Additional file 4 — Directory containing a graphical tool to benchmark new predictions (submitted as input file in the correct format) with the predictions shown in this publication. The graphical tool provided in this compressed archive allows to compute and visualize the results for a new input file containing a new set of predictions. It consist in a web page (index.html) that can be accessed locally or remotely if the directory is placed on a server. The input file is simply drag and dropped on the webpage in order to start the computation of the complete similarity matrix and on the different statistical indicator including the curve of Figure 1. [file 1758-2946-6-9-S4.zip › bestMatchVisualizer/scripts/libs/ckeditor/samples/plugins/magicline/magicline.html]

Using Magicline plugin — CKEditor Sample


# CKEditor Samples » Using Magicline plugin

This sample shows the advantages of **Magicline** plugin
which is to enhance the editing process. Thanks to this plugin,
a number of difficult focus spaces which are inaccessible due to
browser issues can now be focused.

**Magicline** plugin shows a red line with a handler
which, when clicked, inserts a paragraph and allows typing. To see this,
focus an editor and move your mouse above the focus space you want
to access. The plugin is enabled by default so no additional
configuration is necessary.

Editor 1:

This editor uses a default **Magicline** setup.

<table border="1" cellpadding="1" cellspacing="1" style="width: 100%; ">
<tbody>
<tr>
<td>This table</td>
<td>is the</td>
<td>very first</td>
<td>element of the document.</td>
</tr>
<tr>
<td>We are still</td>
<td>able to acces</td>
<td>the space before it.</td>
<td>
<table border="1" cellpadding="1" cellspacing="1" style="width: 100%; ">
<tbody>
<tr>
<td>This table is inside of a cell of another table.</td>
</tr>
<tr>
<td>We can type&nbsp;either before or after it though.</td>
</tr>
</tbody>
</table>
</td>
</tr>
</tbody>
</table>
<p>Two succesive horizontal lines (<tt>HR</tt> tags). We can access the space in between:</p>
<hr />
<hr />
<ol>
<li>This numbered list...</li>
<li>...is a neighbour of a horizontal line...</li>
<li>...and another list.</li>
</ol>
<ul>
<li>We can type between the lists...</li>
<li>...thanks to <strong>Magicline</strong>.</li>
</ul>
<p>Lorem ipsum dolor sit amet dui. Morbi vel turpis. Nullam et leo. Etiam rutrum, urna tellus dui vel tincidunt mattis egestas, justo fringilla vel, massa. Phasellus.</p>
<p>Quisque iaculis, dui lectus varius vitae, tortor. Proin lacus. Pellentesque ac lacus. Aenean nonummy commodo nec, pede. Etiam blandit risus elit.</p>
<p>Ut pretium. Vestibulum rutrum in, adipiscing elit. Sed in quam in purus sem vitae pede. Pellentesque bibendum, urna sem vel risus. Vivamus posuere metus. Aliquam gravida iaculis nisl. Nam enim. Aliquam erat ac lacus tellus ac felis.</p>
<div style="border: 2px dashed green; background: #ddd; text-align: center;">
<p>This text is wrapped in a&nbsp;<tt>DIV</tt>&nbsp;element. We can type after this element though.</p>
</div>

  

Editor 2:

This editor is using a blue line.

```
CKEDITOR.replace( 'editor2', {
	magicline_color: 'blue'
});
```

<table border="1" cellpadding="1" cellspacing="1" style="width: 100%; ">
<tbody>
<tr>
<td>This table</td>
<td>is the</td>
<td>very first</td>
<td>element of the document.</td>
</tr>
<tr>
<td>We are still</td>
<td>able to acces</td>
<td>the space before it.</td>
<td>
<table border="1" cellpadding="1" cellspacing="1" style="width: 100%; ">
<tbody>
<tr>
<td>This table is inside of a cell of another table.</td>
</tr>
<tr>
<td>We can type&nbsp;either before or after it though.</td>
</tr>
</tbody>
</table>
</td>
</tr>
</tbody>
</table>
<p>Two succesive horizontal lines (<tt>HR</tt> tags). We can access the space in between:</p>
<hr />
<hr />
<ol>
<li>This numbered list...</li>
<li>...is a neighbour of a horizontal line...</li>
<li>...and another list.</li>
</ol>
<ul>
<li>We can type between the lists...</li>
<li>...thanks to <strong>Magicline</strong>.</li>
</ul>
<p>Lorem ipsum dolor sit amet dui. Morbi vel turpis. Nullam et leo. Etiam rutrum, urna tellus dui vel tincidunt mattis egestas, justo fringilla vel, massa. Phasellus.</p>
<p>Quisque iaculis, dui lectus varius vitae, tortor. Proin lacus. Pellentesque ac lacus. Aenean nonummy commodo nec, pede. Etiam blandit risus elit.</p>
<p>Ut pretium. Vestibulum rutrum in, adipiscing elit. Sed in quam in purus sem vitae pede. Pellentesque bibendum, urna sem vel risus. Vivamus posuere metus. Aliquam gravida iaculis nisl. Nam enim. Aliquam erat ac lacus tellus ac felis.</p>
<div style="border: 2px dashed green; background: #ddd; text-align: center;">
<p>This text is wrapped in a&nbsp;<tt>DIV</tt>&nbsp;element. We can type after this element though.</p>
</div>

---

CKEditor - The text editor for the Internet - http://ckeditor.com

Copyright © 2003-2013, CKSource - Frederico
Knabben. All rights reserved.
