# Supplementary material for: A new method for the comparison of 1H NMR predictors based on tree-similarity of spectra
Source: J Cheminform. 2014 Mar 25;6:9. doi: 10.1186/1758-2946-6-9 (PMC3987679; doi:10.1186/1758-2946-6-9)
Supplement: Additional file 4 — Directory containing a graphical tool to benchmark new predictions (submitted as input file in the correct format) with the predictions shown in this publication. The graphical tool provided in this compressed archive allows to compute and visualize the results for a new input file containing a new set of predictions. It consist in a web page (index.html) that can be accessed locally or remotely if the directory is placed on a server. The input file is simply drag and dropped on the webpage in order to start the computation of the complete similarity matrix and on the different statistical indicator including the curve of Figure 1. [file 1758-2946-6-9-S4.zip › bestMatchVisualizer/scripts/libs/ckeditor/samples/plugins/toolbar/toolbar.html]

Toolbar Configuration — CKEditor Sample


# CKEditor Samples » Toolbar Configuration

This sample page demonstrates editor with loaded full toolbar (all registered buttons) and, if
current editor's configuration modifies default settings, also editor with modified toolbar.

Since CKEditor 4 there are two ways to configure toolbar buttons.

## By config.toolbar

You can explicitly define which buttons are displayed in which groups and in which order.
This is the more precise setting, but less flexible. If newly added plugin adds its
own button you'll have to add it manually to your `config.toolbar` setting as well.

To add a CKEditor instance with custom toolbar setting, insert the following JavaScript call to your code:

```
CKEDITOR.replace( 'textarea_id', {
	toolbar: [
		{ name: 'document', items: [ 'Source', '-', 'NewPage', 'Preview', '-', 'Templates' ] },	// Defines toolbar group with name (used to create voice label) and items in 3 subgroups.
		[ 'Cut', 'Copy', 'Paste', 'PasteText', 'PasteFromWord', '-', 'Undo', 'Redo' ],			// Defines toolbar group without name.
		'/',																					// Line break - next group will be placed in new line.
		{ name: 'basicstyles', items: [ 'Bold', 'Italic' ] }
	]
});
```

## By config.toolbarGroups

You can define which groups of buttons (like e.g. `basicstyles`, `clipboard`
and `forms`) are displayed and in which order. Registered buttons are associated
with toolbar groups by `toolbar` property in their definition.
This setting's advantage is that you don't have to modify toolbar configuration
when adding/removing plugins which register their own buttons.

To add a CKEditor instance with custom toolbar groups setting, insert the following JavaScript call to your code:

```
CKEDITOR.replace( 'textarea_id', {
	toolbarGroups: [
		{ name: 'document',	   groups: [ 'mode', 'document' ] },			// Displays document group with its two subgroups.
 		{ name: 'clipboard',   groups: [ 'clipboard', 'undo' ] },			// Group's name will be used to create voice label.
 		'/',																// Line break - next group will be placed in new line.
 		{ name: 'basicstyles', groups: [ 'basicstyles', 'cleanup' ] },
 		{ name: 'links' }
	]

	// NOTE: Remember to leave 'toolbar' property with the default value (null).
});
```

## Current toolbar configuration

Below you can see editor with current toolbar definition.

<p>This is some <strong>sample text</strong>. You are using <a href="http://ckeditor.com/">CKEditor</a>.</p>

## Full toolbar configuration

Below you can see editor with full toolbar, generated automatically by the editor.

**Note**: To create editor instance with full toolbar you don't have to set anything.
Just leave `toolbar` and `toolbarGroups` with the default, `null` values.

<p>This is some <strong>sample text</strong>. You are using <a href="http://ckeditor.com/">CKEditor</a>.</p>

---

CKEditor - The text editor for the Internet - http://ckeditor.com

Copyright © 2003-2013, CKSource - Frederico
Knabben. All rights reserved.
