# Supplementary material for: A new method for the comparison of 1H NMR predictors based on tree-similarity of spectra
Source: J Cheminform. 2014 Mar 25;6:9. doi: 10.1186/1758-2946-6-9 (PMC3987679; doi:10.1186/1758-2946-6-9)
Supplement: Additional file 4 — Directory containing a graphical tool to benchmark new predictions (submitted as input file in the correct format) with the predictions shown in this publication. The graphical tool provided in this compressed archive allows to compute and visualize the results for a new input file containing a new set of predictions. It consist in a web page (index.html) that can be accessed locally or remotely if the directory is placed on a server. The input file is simply drag and dropped on the webpage in order to start the computation of the complete similarity matrix and on the different statistical indicator including the curve of Figure 1. [file 1758-2946-6-9-S4.zip › bestMatchVisualizer/scripts/libs/ckeditor/samples/replacebyclass.html]

Replace Textareas by Class Name — CKEditor Sample


# CKEditor Samples » Replace Textarea Elements by Class Name

This sample shows how to automatically replace all `<textarea>` elements
of a given class with a CKEditor instance.

To replace a `<textarea>` element, simply assign it the `ckeditor`
class, as in the code below:

```
<textarea class="ckeditor" name="editor1"></textarea>
```

Note that other `<textarea>` attributes (like `id` or `name`) need to be adjusted to your document.

Editor 1:

<h1><img alt="Saturn V carrying Apollo 11" class="right" src="assets/sample.jpg"/> Apollo 11</h1> <p><b>Apollo 11</b> was the spaceflight that landed the first humans, Americans <a href="http://en.wikipedia.org/wiki/Neil\_Armstrong" title="Neil Armstrong">Neil Armstrong</a> and <a href="http://en.wikipedia.org/wiki/Buzz\_Aldrin" title="Buzz Aldrin">Buzz Aldrin</a>, on the Moon on July 20, 1969, at 20:18 UTC. Armstrong became the first to step onto the lunar surface 6 hours later on July 21 at 02:56 UTC.</p> <p>Armstrong spent about <s>three and a half</s> two and a half hours outside the spacecraft, Aldrin slightly less; and together they collected 47.5 pounds (21.5&nbsp;kg) of lunar material for return to Earth. A third member of the mission, <a href="http://en.wikipedia.org/wiki/Michael\_Collins\_(astronaut)" title="Michael Collins (astronaut)">Michael Collins</a>, piloted the <a href="http://en.wikipedia.org/wiki/Apollo\_Command/Service\_Module" title="Apollo Command/Service Module">command</a> spacecraft alone in lunar orbit until Armstrong and Aldrin returned to it for the trip back to Earth.</p> <h2>Broadcasting and <em>quotes</em> <a id="quotes" name="quotes"></a></h2> <p>Broadcast on live TV to a world-wide audience, Armstrong stepped onto the lunar surface and described the event as:</p> <blockquote><p>One small step for [a] man, one giant leap for mankind.</p></blockquote> <p>Apollo 11 effectively ended the <a href="http://en.wikipedia.org/wiki/Space\_Race" title="Space Race">Space Race</a> and fulfilled a national goal proposed in 1961 by the late U.S. President <a href="http://en.wikipedia.org/wiki/John\_F.\_Kennedy" title="John F. Kennedy">John F. Kennedy</a> in a speech before the United States Congress:</p> <blockquote><p>[...] before this decade is out, of landing a man on the Moon and returning him safely to the Earth.</p></blockquote> <h2>Technical details <a id="tech-details" name="tech-details"></a></h2> <table align="right" border="1" bordercolor="#ccc" cellpadding="5" cellspacing="0" style="border-collapse:collapse;margin:10px 0 10px 15px;"> <caption><strong>Mission crew</strong></caption> <thead> <tr> <th scope="col">Position</th> <th scope="col">Astronaut</th> </tr> </thead> <tbody> <tr> <td>Commander</td> <td>Neil A. Armstrong</td> </tr> <tr> <td>Command Module Pilot</td> <td>Michael Collins</td> </tr> <tr> <td>Lunar Module Pilot</td> <td>Edwin &quot;Buzz&quot; E. Aldrin, Jr.</td> </tr> </tbody> </table> <p>Launched by a <strong>Saturn V</strong> rocket from <a href="http://en.wikipedia.org/wiki/Kennedy\_Space\_Center" title="Kennedy Space Center">Kennedy Space Center</a> in Merritt Island, Florida on July 16, Apollo 11 was the fifth manned mission of <a href="http://en.wikipedia.org/wiki/NASA" title="NASA">NASA</a>&#39;s Apollo program. The Apollo spacecraft had three parts:</p> <ol> <li><strong>Command Module</strong> with a cabin for the three astronauts which was the only part which landed back on Earth</li> <li><strong>Service Module</strong> which supported the Command Module with propulsion, electrical power, oxygen and water</li> <li><strong>Lunar Module</strong> for landing on the Moon.</li> </ol> <p>After being sent to the Moon by the Saturn V&#39;s upper stage, the astronauts separated the spacecraft from it and travelled for three days until they entered into lunar orbit. Armstrong and Aldrin then moved into the Lunar Module and landed in the <a href="http://en.wikipedia.org/wiki/Mare\_Tranquillitatis" title="Mare Tranquillitatis">Sea of Tranquility</a>. They stayed a total of about 21 and a half hours on the lunar surface. After lifting off in the upper part of the Lunar Module and rejoining Collins in the Command Module, they returned to Earth and landed in the <a href="http://en.wikipedia.org/wiki/Pacific\_Ocean" title="Pacific Ocean">Pacific Ocean</a> on July 24.</p> <hr/> <p style="text-align: right;"><small>Source: <a href="http://en.wikipedia.org/wiki/Apollo\_11">Wikipedia.org</a></small></p>

---

CKEditor - The text editor for the Internet - http://ckeditor.com

Copyright © 2003-2013, CKSource - Frederico
Knabben. All rights reserved.
