# Supplementary material for: A new method for the comparison of 1H NMR predictors based on tree-similarity of spectra
Source: J Cheminform. 2014 Mar 25;6:9. doi: 10.1186/1758-2946-6-9 (PMC3987679; doi:10.1186/1758-2946-6-9)
Supplement: Additional file 4 — Directory containing a graphical tool to benchmark new predictions (submitted as input file in the correct format) with the predictions shown in this publication. The graphical tool provided in this compressed archive allows to compute and visualize the results for a new input file containing a new set of predictions. It consist in a web page (index.html) that can be accessed locally or remotely if the directory is placed on a server. The input file is simply drag and dropped on the webpage in order to start the computation of the complete similarity matrix and on the different statistical indicator including the curve of Figure 1. [file 1758-2946-6-9-S4.zip › bestMatchVisualizer/scripts/libs/ckeditor/samples/uicolor.html]

UI Color Picker — CKEditor Sample


# CKEditor Samples » UI Color

This sample shows how to automatically replace `<textarea>` elements
with a CKEditor instance with an option to change the color of its user interface.  
**Note:**The UI skin color feature depends on the CKEditor skin
compatibility. The Moono and Kama skins are examples of skins that work with it.

This editor instance has a UI color value defined in configuration to change the skin color,
To specify the color of the user interface, set the `uiColor` property:

```
CKEDITOR.replace( 'textarea_id', {
	uiColor: '#14B8C4'
});
```

Note that `textarea_id` in the code above is the `id` attribute of
the `<textarea>` element to be replaced.

<p>This is some <strong>sample text</strong>. You are using <a href="http://ckeditor.com/">CKEditor</a>.</p>

---

CKEditor - The text editor for the Internet - http://ckeditor.com

Copyright © 2003-2013, CKSource - Frederico
Knabben. All rights reserved.
