# Supplementary material for: A new method for the comparison of 1H NMR predictors based on tree-similarity of spectra
Source: J Cheminform. 2014 Mar 25;6:9. doi: 10.1186/1758-2946-6-9 (PMC3987679; doi:10.1186/1758-2946-6-9)
Supplement: Additional file 4 — Directory containing a graphical tool to benchmark new predictions (submitted as input file in the correct format) with the predictions shown in this publication. The graphical tool provided in this compressed archive allows to compute and visualize the results for a new input file containing a new set of predictions. It consist in a web page (index.html) that can be accessed locally or remotely if the directory is placed on a server. The input file is simply drag and dropped on the webpage in order to start the computation of the complete similarity matrix and on the different statistical indicator including the curve of Figure 1. [file 1758-2946-6-9-S4.zip › bestMatchVisualizer/scripts/libs/ckeditor/samples/uilanguages.html]

User Interface Globalization — CKEditor Sample


# CKEditor Samples » User Interface Languages

This sample shows how to automatically replace `<textarea>` elements
with a CKEditor instance with an option to change the language of its user interface.

It pulls the language list from CKEditor `_languages.js` file that contains the list of supported languages and creates
a drop-down list that lets the user change the UI language.

By default, CKEditor automatically localizes the editor to the language of the user.
The UI language can be controlled with two configuration options:
`language` and
 `defaultLanguage`. The `defaultLanguage` setting specifies the
default CKEditor language to be used when a localization suitable for user's settings is not available.

To specify the user interface language that will be used no matter what language is
specified in user's browser or operating system, set the `language` property:

```
CKEDITOR.replace( 'textarea_id', {
	// Load the German interface.
	language: 'de'
});
```

Note that `textarea_id` in the code above is the `id` attribute of
the `<textarea>` element to be replaced.

Available languages (  languages!):  
  

(You may see strange characters if your system does not support the selected language)

<p>This is some <strong>sample text</strong>. You are using <a href="http://ckeditor.com/">CKEditor</a>.</p>

---

CKEditor - The text editor for the Internet - http://ckeditor.com

Copyright © 2003-2013, CKSource - Frederico
Knabben. All rights reserved.
