# Supplementary material for: A new method for the comparison of 1H NMR predictors based on tree-similarity of spectra
Source: J Cheminform. 2014 Mar 25;6:9. doi: 10.1186/1758-2946-6-9 (PMC3987679; doi:10.1186/1758-2946-6-9)
Supplement: Additional file 4 — Directory containing a graphical tool to benchmark new predictions (submitted as input file in the correct format) with the predictions shown in this publication. The graphical tool provided in this compressed archive allows to compute and visualize the results for a new input file containing a new set of predictions. It consist in a web page (index.html) that can be accessed locally or remotely if the directory is placed on a server. The input file is simply drag and dropped on the webpage in order to start the computation of the complete similarity matrix and on the different statistical indicator including the curve of Figure 1. [file 1758-2946-6-9-S4.zip › bestMatchVisualizer/scripts/libs/throttle-debounce/docs/files/jquery-ba-throttle-debounce-js.html]

jQuery throttle / debounce: Sometimes, less is more! 


# jQuery throttle / debounce: Sometimes, less is more!

**Version: 1.1, Last updated: 3/7/2010**

|  |  |
| --- | --- |
| Project Home | http://benalman.com/projects/jquery-throttle-debounce-plugin/ |
| GitHub | http://github.com/cowboy/jquery-throttle-debounce/ |
| Source | http://github.com/cowboy/jquery-throttle-debounce/raw/master/jquery.ba-throttle-debounce.js |
| (Minified) | http://github.com/cowboy/jquery-throttle-debounce/raw/master/jquery.ba-throttle-debounce.min.js (0.7kb) |

Summary

|  |  |
| --- | --- |
| jQuery throttle / debounce: Sometimes, less is more! | **Version: 1.1, Last updated: 3/7/2010** |
| License | Copyright © 2010 “Cowboy” Ben Alman, Dual licensed under the MIT and GPL licenses. |
| Examples | These working examples, complete with fully commented code, illustrate a few ways in which this plugin can be used. |
| Support and Testing | Information about what version or versions of jQuery this plugin has been tested with, what browsers it has been tested in, and where the unit tests reside (so you can test it yourself). |
| Release History |  |
| Note for non-jQuery users | jQuery isn’t actually required for this plugin, because nothing internal uses any jQuery methods or properties. |
| Functions |  |
| jQuery.throttle | Throttle execution of a function. |
| jQuery.debounce | Debounce execution of a function. |

### License

Copyright © 2010 “Cowboy” Ben Alman, Dual licensed under the MIT and GPL licenses.  http://benalman.com/about/license/

### Examples

These working examples, complete with fully commented code, illustrate a few ways in which this plugin can be used.

|  |  |
| --- | --- |
| Throttle | http://benalman.com/code/projects/jquery-throttle-debounce/examples/throttle/ |
| Debounce | http://benalman.com/code/projects/jquery-throttle-debounce/examples/debounce/ |

### Support and Testing

Information about what version or versions of jQuery this plugin has been tested with, what browsers it has been tested in, and where the unit tests reside (so you can test it yourself).

|  |  |
| --- | --- |
| jQuery Versions | none, 1.3.2, 1.4.2 |
| Browsers Tested | Internet Explorer 6-8, Firefox 2-3.6, Safari 3-4, Chrome 4-5, Opera 9.6-10.1. |
| Unit Tests | http://benalman.com/code/projects/jquery-throttle-debounce/unit/ |

### Release History

|  |  |
| --- | --- |
| 1.1 | (3/7/2010) Fixed a bug in jQuery.throttle where trailing callbacks executed later than they should.  Reworked a fair amount of internal logic as well. |
| 1.0 | (3/6/2010) Initial release as a stand-alone project.  Migrated over from jquery-misc repo v0.4 to jquery-throttle repo v1.0, added the no\_trailing throttle parameter and debounce functionality. |

### Note for non-jQuery users

jQuery isn’t actually required for this plugin, because nothing internal uses any jQuery methods or properties. jQuery is just used as a namespace under which these methods can exist.

Since jQuery isn’t actually required for this plugin, if jQuery doesn’t exist when this plugin is loaded, the method described below will be created in the `Cowboy` namespace.  Usage will be exactly the same, but instead of $.method() or jQuery.method(), you’ll need to use Cowboy.method().

### Functions

### jQuery.throttle

Throttle execution of a function.  Especially useful for rate limiting execution of handlers on events like resize and scroll.  If you want to rate-limit execution of a function to a single time, see the jQuery.debounce method.

In this visualization, | is a throttled-function call and X is the actual callback execution:

> ```
> Throttled with `no_trailing` specified as false or unspecified:
> ||||||||||||||||||||||||| (pause) |||||||||||||||||||||||||
> X    X    X    X    X    X        X    X    X    X    X    X
>
> Throttled with `no_trailing` specified as true:
> ||||||||||||||||||||||||| (pause) |||||||||||||||||||||||||
> X    X    X    X    X             X    X    X    X    X
> ```

#### Usage

> ```
> var throttled = jQuery.throttle( delay, [ no_trailing, ] callback );
>
> jQuery('selector').bind( 'someevent', throttled );
> jQuery('selector').unbind( 'someevent', throttled );
> ```

#### This also works in jQuery 1.4+

> ```
> jQuery('selector').bind( 'someevent', jQuery.throttle( delay, [ no_trailing, ] callback ) );
> jQuery('selector').unbind( 'someevent', callback );
> ```

#### Arguments

|  |  |
| --- | --- |
| delay | (Number) A zero-or-greater delay in milliseconds.  For event callbacks, values around 100 or 250 (or even higher) are most useful. |
| no\_trailing | (Boolean) Optional, defaults to false.  If no\_trailing is true, callback will only execute every `delay` milliseconds while the throttled-function is being called.  If no\_trailing is false or unspecified, callback will be executed one final time after the last throttled-function call.  (After the throttled-function has not been called for `delay` milliseconds, the internal counter is reset) |
| callback | (Function) A function to be executed after delay milliseconds.  The `this` context and all arguments are passed through, as-is, to `callback` when the throttled-function is executed. |

#### Returns

(Function) A new, throttled, function.

### jQuery.debounce

Debounce execution of a function.  Debouncing, unlike throttling, guarantees that a function is only executed a single time, either at the very beginning of a series of calls, or at the very end.  If you want to simply rate-limit execution of a function, see the jQuery.throttle method.

In this visualization, | is a debounced-function call and X is the actual callback execution:

> ```
> Debounced with `at_begin` specified as false or unspecified:
> ||||||||||||||||||||||||| (pause) |||||||||||||||||||||||||
>                          X                                 X
>
> Debounced with `at_begin` specified as true:
> ||||||||||||||||||||||||| (pause) |||||||||||||||||||||||||
> X                                 X
> ```

#### Usage

> ```
> var debounced = jQuery.debounce( delay, [ at_begin, ] callback );
>
> jQuery('selector').bind( 'someevent', debounced );
> jQuery('selector').unbind( 'someevent', debounced );
> ```

#### This also works in jQuery 1.4+

> ```
> jQuery('selector').bind( 'someevent', jQuery.debounce( delay, [ at_begin, ] callback ) );
> jQuery('selector').unbind( 'someevent', callback );
> ```

#### Arguments

|  |  |
| --- | --- |
| delay | (Number) A zero-or-greater delay in milliseconds.  For event callbacks, values around 100 or 250 (or even higher) are most useful. |
| at\_begin | (Boolean) Optional, defaults to false.  If at\_begin is false or unspecified, callback will only be executed `delay` milliseconds after the last debounced-function call.  If at\_begin is true, callback will be executed only at the first debounced-function call.  (After the throttled-function has not been called for `delay` milliseconds, the internal counter is reset) |
| callback | (Function) A function to be executed after delay milliseconds.  The `this` context and all arguments are passed through, as-is, to `callback` when the debounced-function is executed. |

#### Returns

(Function) A new, debounced, function.

Generated by Natural Docs

jQuery throttle / debounce

Index

Everything

Files

Functions

EverythingFilesFunctions


Throttle execution of a function.

Debounce execution of a function.

Close
