# Supplementary material for: A new method for the comparison of 1H NMR predictors based on tree-similarity of spectra
Source: J Cheminform. 2014 Mar 25;6:9. doi: 10.1186/1758-2946-6-9 (PMC3987679; doi:10.1186/1758-2946-6-9)
Supplement: Additional file 4 — Directory containing a graphical tool to benchmark new predictions (submitted as input file in the correct format) with the predictions shown in this publication. The graphical tool provided in this compressed archive allows to compute and visualize the results for a new input file containing a new set of predictions. It consist in a web page (index.html) that can be accessed locally or remotely if the directory is placed on a server. The input file is simply drag and dropped on the webpage in order to start the computation of the complete similarity matrix and on the different statistical indicator including the curve of Figure 1. [file 1758-2946-6-9-S4.zip › bestMatchVisualizer/scripts/libs/throttle-debounce/docs/index/Functions.html]

Function Index 


Function Index

$#! · 0-9 · A · B · C · D · E · F · G · H · I · J · K · L · M · N · O · P · Q · R · S · T · U · V · W · X · Y · Z

|  |  |
| --- | --- |
| D |  |
|  | debounce, jQuery |
| T |  |
|  | throttle, jQuery |

Debounce execution of a function.


Throttle execution of a function.

Generated by Natural Docs

jQuery throttle / debounce

Index

Everything

Files

Functions

EverythingFilesFunctions

Close
