# Supplementary material for: A new method for the comparison of 1H NMR predictors based on tree-similarity of spectra
Source: J Cheminform. 2014 Mar 25;6:9. doi: 10.1186/1758-2946-6-9 (PMC3987679; doi:10.1186/1758-2946-6-9)
Supplement: Additional file 4 — Directory containing a graphical tool to benchmark new predictions (submitted as input file in the correct format) with the predictions shown in this publication. The graphical tool provided in this compressed archive allows to compute and visualize the results for a new input file containing a new set of predictions. It consist in a web page (index.html) that can be accessed locally or remotely if the directory is placed on a server. The input file is simply drag and dropped on the webpage in order to start the computation of the complete similarity matrix and on the different statistical indicator including the curve of Figure 1. [file 1758-2946-6-9-S4.zip › bestMatchVisualizer/scripts/libs/throttle-debounce/docs/index/General.html]

Index 


Index

$#! · 0-9 · A · B · C · D · E · F · G · H · I · J · K · L · M · N · O · P · Q · R · S · T · U · V · W · X · Y · Z

|  |  |
| --- | --- |
| D |  |
|  | debounce, jQuery |
| E |  |
|  | Examples |
| F |  |
|  | Functions |
| J |  |
|  | jQuery throttle/debounce:Sometimes,less is more! |
| L |  |
|  | License |
| N |  |
|  | Note for non-jQuery users |
| R |  |
|  | Release History |
| S |  |
|  | Support and Testing |
| T |  |
|  | throttle, jQuery |

Debounce execution of a function.


These working examples, complete with fully commented code, illustrate a few ways in which this plugin can be used.


**Version: 1.1, Last updated: 3/7/2010**


Copyright © 2010 “Cowboy” Ben Alman, Dual licensed under the MIT and GPL licenses.


jQuery isn’t actually required for this plugin, because nothing internal uses any jQuery methods or properties.


Information about what version or versions of jQuery this plugin has been tested with, what browsers it has been tested in, and where the unit tests reside (so you can test it yourself).


Throttle execution of a function.

Generated by Natural Docs

jQuery throttle / debounce

Index

Everything

Files

Functions

EverythingFilesFunctions

Close
